# Accelerating the Design of Self-Guided Microrobots in Time-Varying Magnetic Fields

**DOI:** 10.1021/jacsau.2c00499

**Published:** 2023-03-10

**Authors:** Kiran Dhatt-Gauthier, Dimitri Livitz, Yiyang Wu, Kyle J. M. Bishop

**Affiliations:** Department of Chemical Engineering, Columbia University, New York, New York 10027, United States

**Keywords:** autonomous, microbot, physical intelligence, active colloids, magnetic actuation, stimuli-responsive

## Abstract

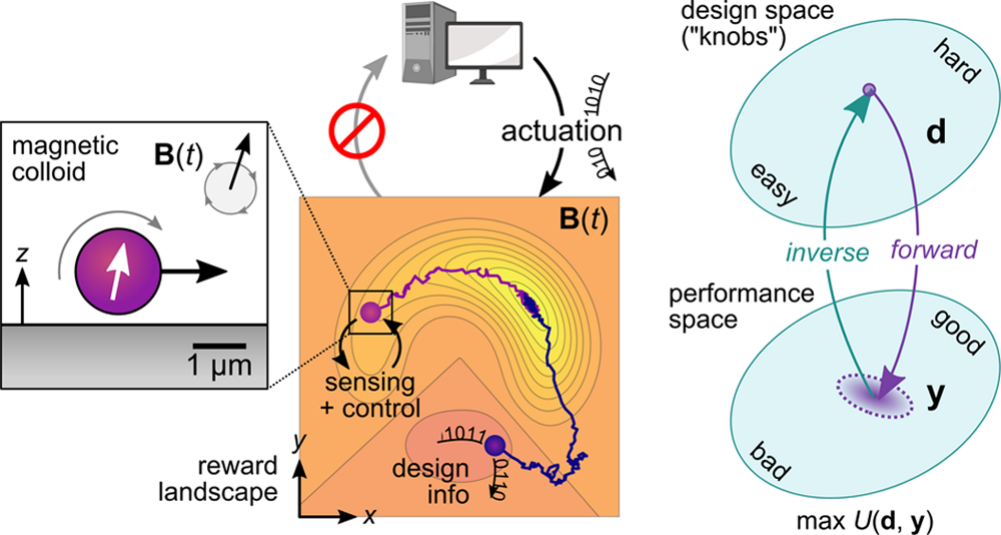

Mobile robots combine sensory information with mechanical actuation to move autonomously through structured environments and perform specific tasks. The miniaturization of such robots to the size of living cells is actively pursued for applications in biomedicine, materials science, and environmental sustainability. Existing microrobots based on field-driven particles rely on knowledge of the particle position and the target destination to control particle motion through fluid environments. Often, however, these external control strategies are challenged by limited information and global actuation where a common field directs multiple robots with unknown positions. In this Perspective, we discuss how time-varying magnetic fields can be used to encode the self-guided behaviors of magnetic particles conditioned on local environmental cues. Programming these behaviors is framed as a design problem: we seek to identify the design variables (e.g., particle shape, magnetization, elasticity, stimuli-response) that achieve the desired performance in a given environment. We discuss strategies for accelerating the design process using automated experiments, computational models, statistical inference, and machine learning approaches. Based on the current understanding of field-driven particle dynamics and existing capabilities for particle fabrication and actuation, we argue that self-guided microrobots with potentially transformative capabilities are close at hand.

## 1 Introduction

Inspired by living cells, the development of mobile robots on the micron scale promises new capabilities for advancing human health, renewable energy, and environmental sustainability.^1−8^ Owing to their small size, such microrobots are capable of navigating through structured environments that are otherwise inaccessible—for example, through biological tissues, battery materials, groundwater aquifers, etc. Within these environments, it is envisioned that microrobots could be programmed to perform desired functions involving the localized manipulation of information, matter, and energy. They should be capable of sensing, recording, and transmitting information about their microenvironment for precision diagnostics. They should be able to capture material cargo, transport it to targeted locations, and release it on demand for therapeutic applications. They should exert stresses, emit light, and/or generate heat so as to alter the local microstructure for applications in microsurgery and material repair. Ultimately, synthetic microrobots aim to reproduce the functional capabilities of living cells while operating also in extreme environments hostile to life. In one imagined scenario, microrobots incorporated within the electrolyte of a lithium metal battery patrol the electrode surface in search of lithium dendrites which they eliminate to prevent short circuits and prolong battery life. Despite recent progress in our understanding and control of self-propelled microparticles,^2,9^ the majority of these capabilities remain beyond the reach of current technologies. These limitations are particularly severe for *self-guided* robots that operate autonomously without external control systems.

**Figure 1:**
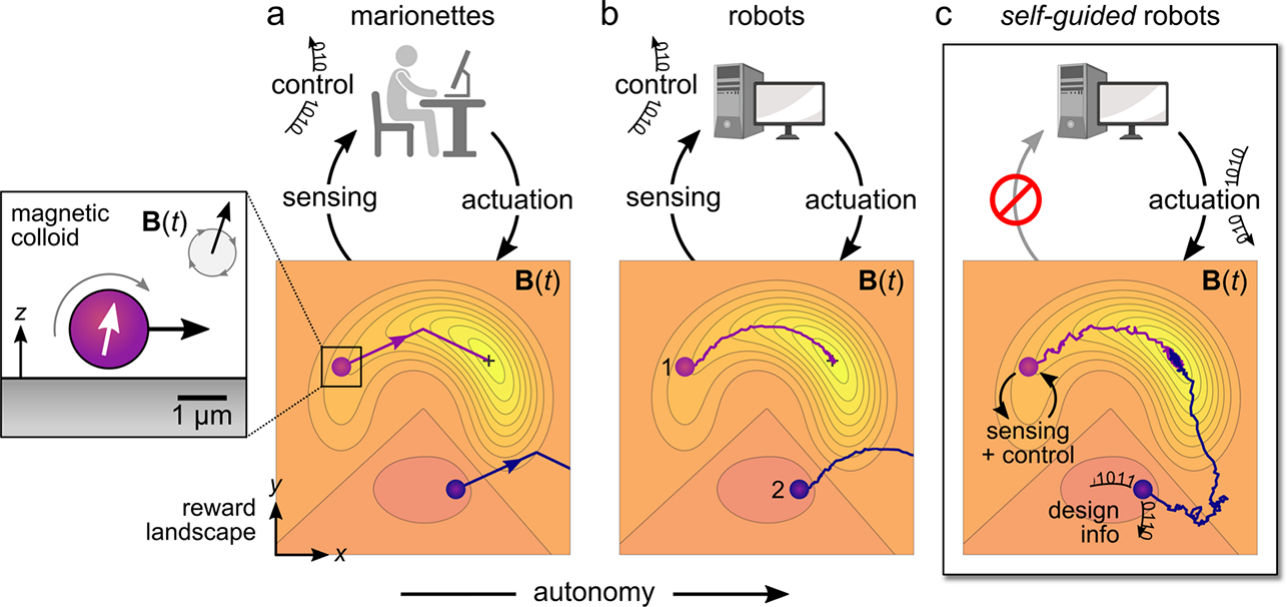
Schematic illustration of increasingly autonomous microrobots moving on a user-defined reward landscape. These “robots” are simply magnetic particles that roll across a solid surface in a time-varying magnetic field **B**(*t*). (a) With knowledge of the particle position, a human controller can direct the motion of a single *marionette*(13) to a desired location; a second particle experiences the same field and moves to an undesired location. (b) Using real-time feedback between the particle position (e.g., from microscopy) and the applied field, a computer-based control system can direct the autonomous migration of a single *robot* to the target location. (c) *Self-guided* robots use internal mechanisms of sensing and control to navigate the reward landscape in a common time-varying field. The physical intelligence^14^ of these systems is encoded in the design of the particle and the driving field (see Section 3.1 for a concrete example based on topotaxis^15^).

To illustrate the challenge, consider a primitive robot based on a magnetic colloid driven to roll on a surface by a time-varying magnetic field **B**(*t*) (Figure 1). The goal of this robot is to navigate a 2D reward landscape in pursuit of a local maximum—analogous to a chemotactic bacterium in pursuit of chemical fuel. This simple task can be achieved with different levels of autonomy.^10^ Using knowledge of the particle position, a human controller can determine the orientation of a rotating field that directs the magnetic roller up the reward landscape (Figure 1a). Greater autonomy is achieved by replacing the human with a computer-based controller using the same sensors and actuators (Figure 1b).^11^ In the absence of real-time sensing, planning algorithms based on prior knowledge of the landscape and predictive models of the robot dynamics can be used to identify effective actuation schedules.^12^ In each case, the robot’s behavior is determined by sensors and controllers *external* to the robot itself—for example, a human operator with an optical microscope.

By contrast, self-guided robots use *internal* mechanisms of sensing and control to enable autonomous navigation of structured environments without knowledge of the robot’s position (Figure 1c). Robot vacuum cleaners and self-driving cars provide familiar examples at the macroscale; however, it remains challenging to miniaturize these technologies to the microscopic dimensions of living cells. Instead, current microrobots achieve primitive forms of sensing and control based on their physicochemical dynamics and the interactions with their environment.^16^ This type of “physical intelligence” at the microscale provides a feasible alternative to the “computational intelligence” of macroscopic robots.^14^ Continuing the example above, the dynamics of the magnetic roller is sensitive to the proximity and orientation of a nearby surface. These hydrodynamic interactions provide a basis for self-guided navigation across topographic landscapes—so-called topotaxis^15^—in which the desired behavior is encoded in the relevant design variables that influence the robot’s dynamics. The goal of this Perspective is to identify strategies for accelerating the design of self-guided microbots that exhibit increasing levels of physical intelligence.

We focus our discussion on a particular class of microrobots—namely, those powered and directed by external magnetic fields.^17−21^ Currently, these magnetic microrobots are little more than colloidal particles that swim, roll, or crawl through fluid environments under the influence of time-varying fields. Such fields are not scattered or screened by common materials (e.g., human tissue) and can therefore act remotely and specifically to actuate magnetic particles introduced for that purpose. The physics of magnetic actuation and propulsion is well understood and can therefore be used to accelerate the design of microrobots for targeted functions. We consider application contexts characterized by global actuation and limited information, in which a common time-varying field directs the operation of multiple robots with unknown positions. In this context, the functional behavior of each microrobot is determined both by its local environment and by the common field. For example, microrobots dispersed in the bloodstream might use local hydrodynamic cues like the fluid velocity and its gradient to direct their self-guided navigation.

**Figure 2:**
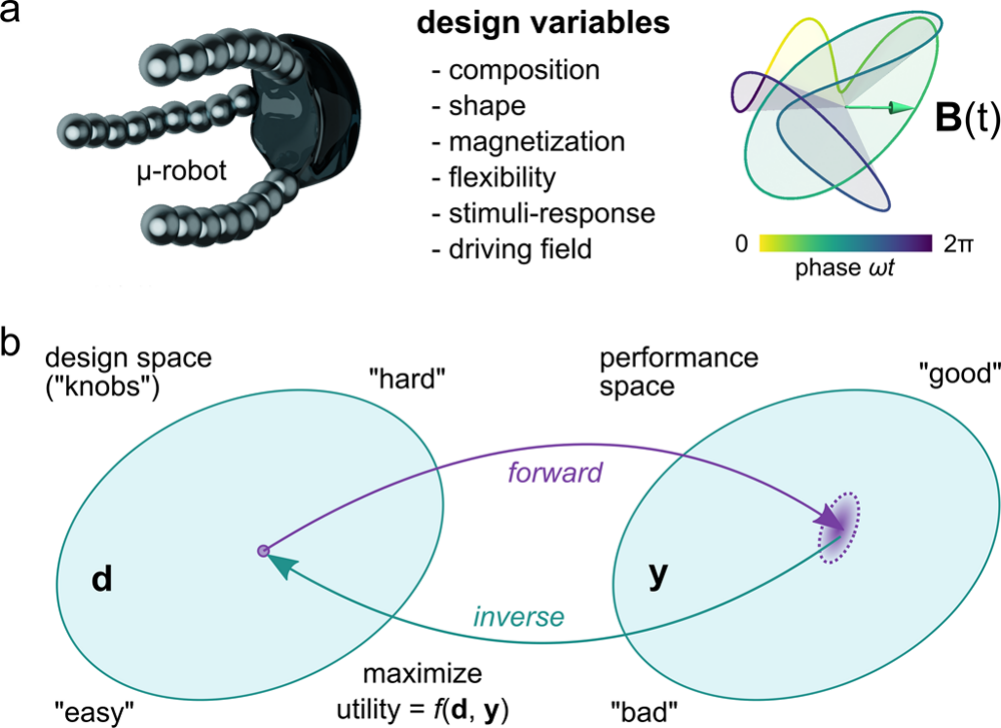
(a) The self-guided capabilities of magnetic microrobots are encoded in their material properties (e.g., shape, magnetization, flexibility, stimuli-response) and in the waveform of the time-varying magnetic field **B**(*t*). The selection of these design variables (“knobs”) determines the dynamics of the robot in a specified environment as discussed in Section 2. (b) Programming a self-guided robot can be framed as a design problem: we seek design variables and performance metrics that maximize the expected utility, which accounts for both design costs and performance benefits. The design process can be accelerated using computational models, automated experiments, statistical inference, and machine learning as discussed in Section 3.

Encoding the behaviors of self-guided microrobots can be framed as a design problem (Figure 2). By carefully selecting the relevant design variables—for example, the waveform of the driving field, the shape of the magnetic particle—one seeks targeted behaviors that can be quantified by suitable performance metrics. In the context of self-guided navigation, we seek microrobots that move with a desired speed and direction in response to local gradients in their environment (i.e., taxis). This design problem is challenging since the relationship between microrobot design and performance is often high-dimensional, nonlinear, stochastic, and unknown. Informed by experimental data, predictive models can provide useful approximations to these relationships that guide the search for better designs. By contrast, life’s microrobots were designed by a blind process of evolution by natural selection, which relies on long time scales and a massively parallel search to achieve their remarkable capabilities. This difference begs the question: what can we (humans) hope to achieve using intelligent design^22^ over decades compared to life’s marvels forged by eons of evolutionary design work? Mindful of Orgel’s rule that “evolution is cleverer than you are”, the answer could be disappointing. Nevertheless, we are optimistic that recent advances in computation, automation, and machine learning can significantly accelerate the design of self-guided microrobots with useful—albeit primitive—capabilities.

This Perspective is divided in two parts: the “forward problem” of exploring the dynamics of magnetic microrobots afforded by increasingly complex design spaces, and the “inverse problem” of identifying those designs that enable self-guided behaviors (Figure 2b). In part one, we review the physics of magnetic actuation and describe how spatiotemporal fields are used to position and propel magnetic particles in viscous fluids. We highlight examples from literature that illustrate the relevant design variables and their impact on particle dynamics and robot capabilities. In part two, we discuss how predictive models trained and validated on experimental data can be used to accelerate the design of self-guided behaviors. In particular, we highlight the use of complex time-varying fields for encoding gradient-driven taxis of magnetic particles. The design of these and other functions will require the close integration of automated experiments, computer simulations, statistical inference, and machine learning approaches. We outline strategies for navigating the growing space of possible designs in pursuit of self-guided microrobots that respond intelligently to an growing number of environmental stimuli.

## 2 Forward Problem: Understanding Microrobot Dynamics

External magnetic fields can be used to position, propel, and deform micron-scale particles immersed in viscous fluid environments. The dynamics of these primitive microrobots depends on the particle’s magnetic response and its interactions with the external field and the surrounding fluid (see Supporting Information, Section 2). In this brief review, we describe how external fields and their gradients can be used to specify the position and orientation of a magnetic particle in three dimensions. We discuss how time-varying fields can be used to propel motion at low Reynolds numbers by coupling rotation and translation using asymmetries in the particle shape or its environment. Beyond the field-driven dynamics of rigid bodies, we consider the actuation of deformable particle assemblies held together by elastic, magnetic, and/or hydrodynamic interactions. Overall, this section highlights the many design variables by which to control particle dynamics and thereby the self-guided capabilities of magnetic microrobots.

### 2.1 Particle Positioning

**Figure 3:**
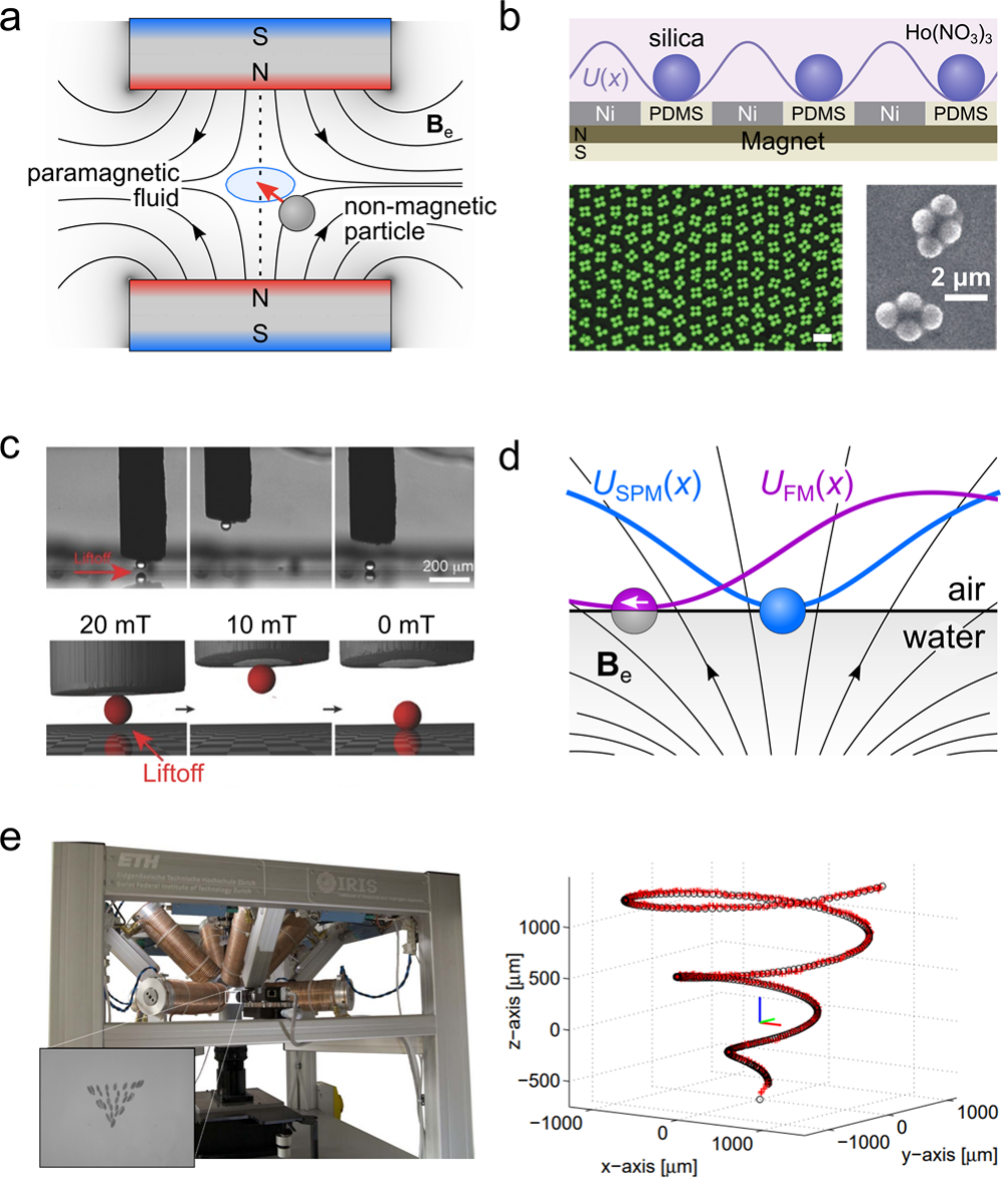
(a) A nonmagnetic particle suspended in paramagnetic fluid levitates between two permanent magnets where the field magnitude is minimal (so-called magnetic levitation or MagLev).^24^ (b) Patterned magnetic surfaces direct the assembly of nonmagnetic colloids in paramagnetic solutions of holmium nitrate, reproduced with permission from ref (27). Copyright (2013) Springer Nature. (c) Magnetophoretic “tweezers” use micron-scale probes to manipulate magnetic and nonmagnetic objects with local field gradients.^29^ Shown here is the capture and release of a 50 μm silica sphere in a paramagnetic fluid.^30^ Reproduced with permission from ref (30). Copyright (2016) WILEY-VCH Verlag GmbH & Co. KGaA, Weinheim. (d) Ferromagnetic (FM, purple) and superparamagnetic (SPM, blue) particles adsorbed at a liquid interface adopt stable positions in a nonuniform field as to minimize the magnetic energy *U*(*x*). Here, the moment of the ferromagnetic Janus sphere is directed parallel to the interface.^31,32^ (e) The OctoMag system (left) uses eight electromagnets to specify the field and its gradient at the site of a ferromagnetic particle.^33^ Using knowledge of the particle position in 3D, control algorithms enable the directed motion of the particle along prescribed trajectories (right). Reproduced with permission from ref (33). Copyright (2010) IEEE.

One of the basic challenges of microrobotics is controlling the position and orientation of a magnetic particle in three-dimensions. Arguably, the simplest approach uses structured magnetic fields to achieve the passive levitation of nonmagnetic particles in a paramagnetic fluid. For example, the field produced by two permanent magnets in the anti-Helmholtz configuration creates a bowl-shaped potential well that specifies the equilibrium particle position (Figure 3a).^23,24^ Typically, magnetic levitation experiments are conducted in aqueous solutions of paramagnetic salts; however, stronger trapping forces can be achieved using ferrofluids.^25,26^ By structuring the field using patterned magnet arrays, one can shape the energy landscape to create “magnetic molds” that control the positions and orientations of dispersed microparticles (Figure 3b).^27,28^ A major limitation of this approach, however, is its reliance on magnetic media, which are rarely encountered in the context of robotic applications (e.g., in the human body).

To position magnetic microparticles in nonmagnetic media, different strategies are required to account for the inevitable attraction of magnetic particles to high field regions (see Supporting Information, Section 2). One approach uses micron-scale probes—so-called magnetophoretic tweezers—to generate local field gradients that capture nearby particles and move them using micropositioners (Figure 3c).^29,30^ Alternatively, magnetic particles can be adsorbed onto a fluid interface such that field gradients in three dimensions create local energy minima for particle positioning in two dimensions (Figure 3d).^34−36^ At *curved* interfaces, even uniform fields can be used to position ferromagnetic particles due to coupling between magnetic and capillary torques.^31,32^ Achieving full control at-a-distance over the position and orientation of a single magnetic particle in three dimensions requires multiple electromagnetics with which to specify the field and its gradient at the particle location (Figure 3e, left).^33^ Using real-time knowledge of the particle position, feedback control algorithms direct the tuning of the electromagnets to guide particle motion to a targeted location or along specified trajectories (Figure 3e, right).^33^ Despite these impressive capabilities, it remains challenging to control the particle when its position is unknown (limited information) or to position multiple particles independently using a common field (global actuation).

### 2.2 Torque-Driven Propulsion

To drive the rapid propulsion of micron-scale colloids using external magnetic fields, there are significant advantages to using magnetic torques in spatially uniform fields as compared to magnetic forces due to field gradients. A simple scaling argument reveals why. The characteristic speed of a ferromagnetic sphere with diameter *d* and moment *m* in a magnetic field of strength *B*_e_ that decays over length *L* is *U* ∼ *mB*_e_/3*πηdL*, obtained by balancing the magnetic force and the viscous drag. A uniform field can rotate the same particle at angular speeds of order Ω ∼ *mB*_e_/*πηd*^3^ by a similar argument. Perfect coupling between particle rotation and translation—for example, frictional rolling on a solid surface—would lead to propulsion speeds of order *U* ∼ *mB*_e_/2*πηd*^2^, which exceeds that due to field gradients by a large factor of *L*/*d* ≫ 1. In practice, rotation–translation coupling with a nearby surface is weaker due to hydrodynamic “slipping”; however, the same argument applies. Even far from solid boundaries, asymmetric particle shapes can enable steady propulsion in uniform time-varying fields due to hydrodynamic coupling between rotation and translation. Here, we briefly review these and other modes of magnetic particle propulsion relevant to the realization of magnetic microrobots.

#### 2.2.1 Free Swimmers

We first consider the field-driven propulsion of “free swimmers”—that is, magnetic particles that move through an unbounded fluid far from solid boundaries (and each other). In the simplest case of a *rigid* ferromagnetic particle, the dynamics is described by a combination of magnetic actuation and low-Reynolds number hydrodynamics. The characteristic Reynolds number for micron-scale particles rotating in water due to 1 mT fields is Re = *ρd*^2^Ω/η ∼ 10^–3^ ≪ 1, which implies that inertial effects are negligible. In a quiescent fluid, the linear and angular velocity of the particle are linearly related to the magnetic force and torque by the hydrodynamic mobility tensor, which depends on the size, shape, and orientation of the particle as well as its position relative to nearby boundaries.^37,38^ For asymmetric particles in an unbounded fluid, field-driven rotation can propel linear motion due to nonzero coupling between particle rotation and translation.^39^

**Figure 4:**
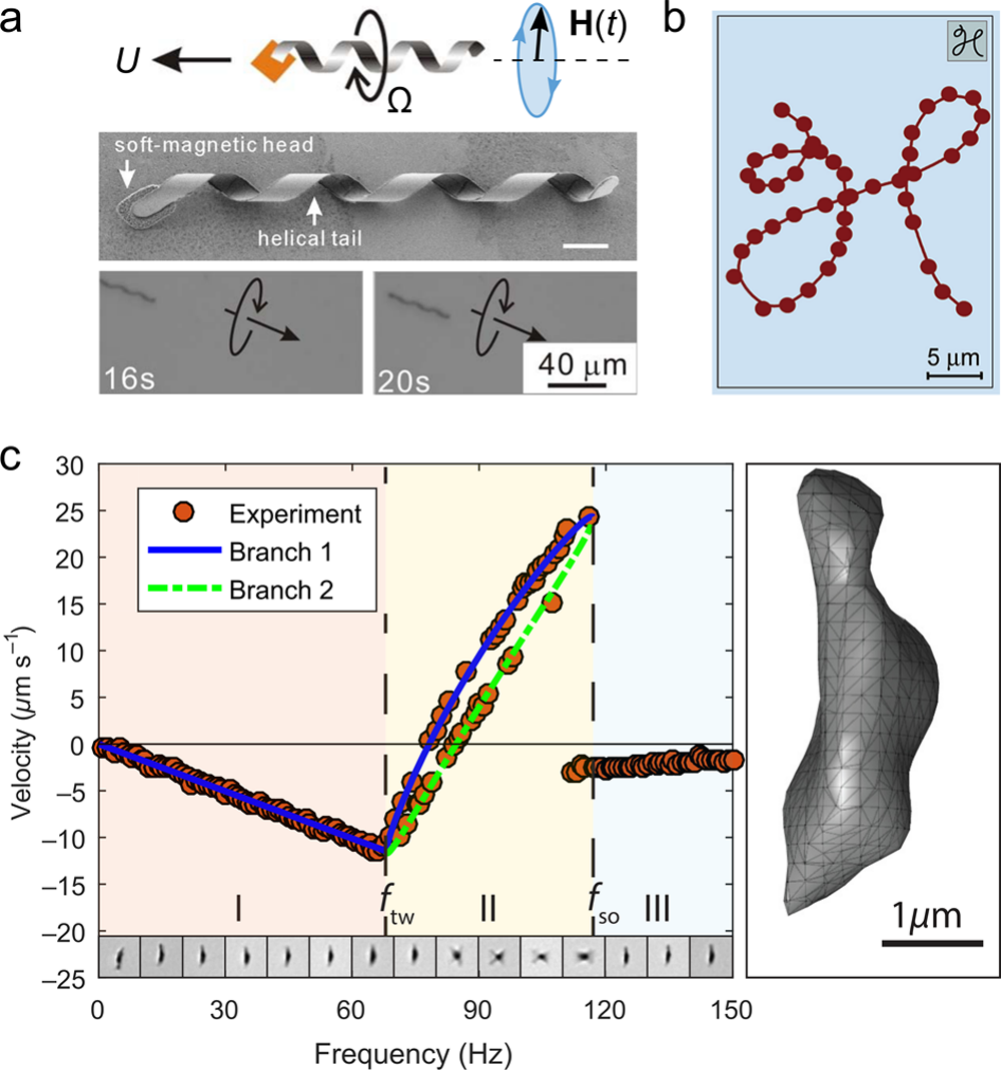
(a) The rotation of helical particles in a rotating field leads to directed translation due to hydrodynamic coupling between rotation and translation. Reproduced with permission from ref (40). Copyright (2009) AIP Publishing. (b) Using time-varying fields, the trajectory of a helical swimmer can be directed along complex preprogrammed paths. Reproduced with permission from ref (41). Copyright (2009) American Chemical Society. (c) The velocity of a ferromagnetic particle of irregular shape exhibits dynamical transitions with increasing frequency of a rotating magnetic field.^42^ The particle’s 3D shape (right) is reconstructed from optical microscopy images. Reproduced with permission from ref (42). Copyright (2019) American Physical Society.

The speed of magnetic propulsion depends sensitively on particle shape as well as the frequency and waveform of the time-varying field. The canonical example is a long helical particle with a permanent magnetic moment oriented perpendicular to its axis (Figure 4a,b).^17,40,41^ Application of a rotating field causes the particle to rotate and translate in a direction specified by the external field and the particle chirality. In this way, microrobots can be driven to trace complex trajectories in three dimensions^41^ and to transport colloidal cargo using microholders.^43^ At low frequencies, the particle rotates in synchrony with the applied field, thereby “screwing” through the fluid at a constant speed. Importantly, the propulsion speed depends on particle shape—for example, on the radius and pitch of the helix. While some shapes are more effective than others, any low symmetry particle with a magnetic moment will swim along the axis of the rotating field (Figure 4c).^42,44^ With increasing rotation frequency, particles exhibit one or more bifurcations in their rotational dynamics that alter the speed—and sometimes the direction—of propulsion (Figure 4c). The nonlinear dynamics governing the orientation of low symmetry particles in 3D is nontrivial and allows for multiple stable solutions. For example, the asymmetric particle in Figure 4c shows two stable modes of rotation–translation in a common rotating field at intermediate frequencies. As discussed in Section 2.3 below, additional types of free swimming become possible for *flexible* particles with internal degrees of freedom.

#### 2.2.2 Surface Rollers

**Figure 5:**
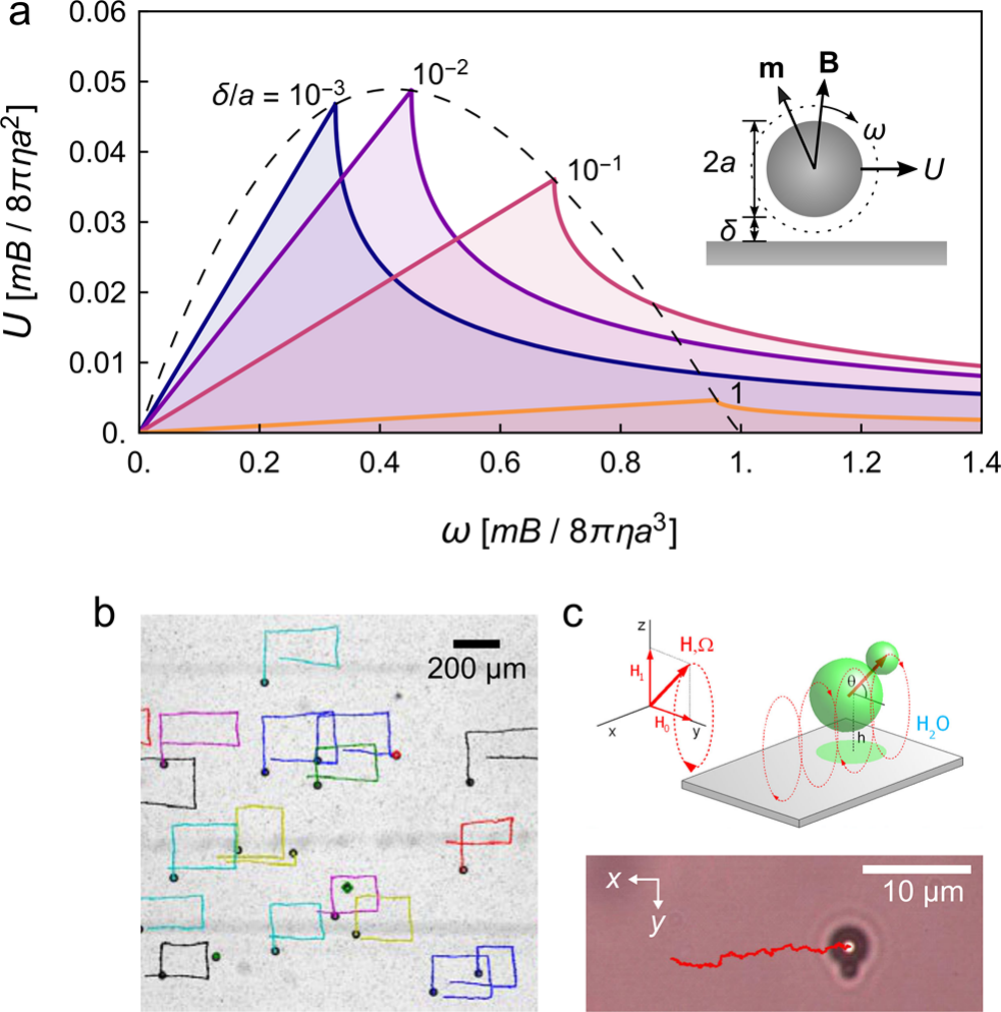
(a) Predicted rolling speed *U* for a ferromagnetic sphere in a rotating magnetic field with frequency ω. The different curves correspond to different surface separations δ. See Supporting Information for details. (b) Multiple ferromagnetic rollers trace rectangular trajectories on a solid substrate as directed by a rotating field with changing orientation. Reproduced with permission from ref (45). Copyright (2017) Springer Nature. (c) Superparamagnetic particles of asymmetric shape (here, a two sphere doublet) translate across along a solid substrate in a precessing field. Reproduced with permission from ref (48). Copyright (2008) American Physical Society.

The presence of nearby boundaries introduces new mechanisms for rotation-translation coupling that enable torque-driven propulsion of high symmetry particles. Figure 5a illustrates the specific case of a ferromagnetic sphere “rolling” across a planar substrate as directed by a rotating magnetic field.^15,45^ The particle speed increases linearly with rotation frequency up to some critical value, above which the hydrodynamic resistance to rotation exceeds the magnetic torque. At higher frequencies (ω > ω_c_), repeated “slipping” of the moment relative to the field leads to fluctuating torques that drive particle translation at slower speeds. Notably, this hydrodynamic model suggests that the maximum speed *U*_max_ and the critical frequency ω_c_ depend on the surface separation δ (Figure 5a, dashed curve). Decreasing this separation—for example, using gravitational or magnetic forces—can act to strengthen rotation-translation coupling thereby enhancing propulsion at constant frequency ω < ω_c_.^46^ Small separations, however, also increase the resistance to rotation thereby decreasing the critical frequency ω_c_. These competing effects lead to a maximum propulsion speed at surface separations equal to ca. 1% of the particle diameter (Figure 5a). By changing the orientation of the rotating field, multiple spheres can trace complex trajectories across the 2D surface (Figure 5b).^45^ Such magnetic rollers provide a basis for microrobots that move along blood vessel walls to perform theranostic functions within the human vasculature.^47^

Superparamagnetic particles can also be driven to “roll” on surfaces but require anisotropic polarizability or high frequency fields to induce the necessary torques. Figure 5c shows one example in which an asymmetric colloidal doublet translates across a surface under the influence of a precessing magnetic field.^48^ At sufficiently high frequencies, even superparamagnetic spheres with isotropic polarizability can be driven roll due to the finite time scale of magnetization.^49,50^ Application of a rotating field creates a time-averaged magnetic torque that reaches its maximum value when the angular frequency equals the relaxation rate (see Supporting Information, Section 4).

In the examples above, the direction of particle motion is dictated by that of the applied field (see, for example, Figure 5b). To enable *self-guided* propulsion in the plane, driving fields of higher symmetry are required—for example, an oscillating field normal to the surface. Such fields can power particle propulsion along any direction of a solid substrate due to asymmetric particle shapes^51^ or spontaneous symmetry breaking.^52^ For example, ferromagnetic spheres with non-negligible inertia (e.g., 60 μm Ni) break axial symmetry and roll across a solid surface in an oscillating field of appropriate magnitude and frequency.^52^ While such motion is prohibited for smaller microspheres in viscous fluids, anisotropic particles can also be driven to swim (not necessarily roll) across planar surfaces in linearly oscillating fields.^51^ Alternatively, the asymmetry necessary for propulsion at low Reynolds number can be introduced using the surface topography of the underlying substrate.^53^

### 2.3 Shape-Changing Microrobots

To achieve increasingly complex tasks, magnetic microrobots benefit from internal degrees of freedom that enable new behaviors conditioned on the current state of the robot.^54^ For rigid particles discussed in the previous section, the robot’s dynamical state is described by only few variables—for example, the orientation of a ferromagnetic sphere and its height above a plane wall. Connecting multiple particles together using flexible linkers or other interparticle interactions provides a basis for shape-changing robots that can adopt many possible configurations to achieve new modes of propulsion, cargo capture-transport-release, and the ability to assemble–disassemble on demand. In this section, we discuss some illustrative examples of composite, multiparticle robots, and their capabilities.

#### 2.3.1 1D Chains

**Figure 6:**
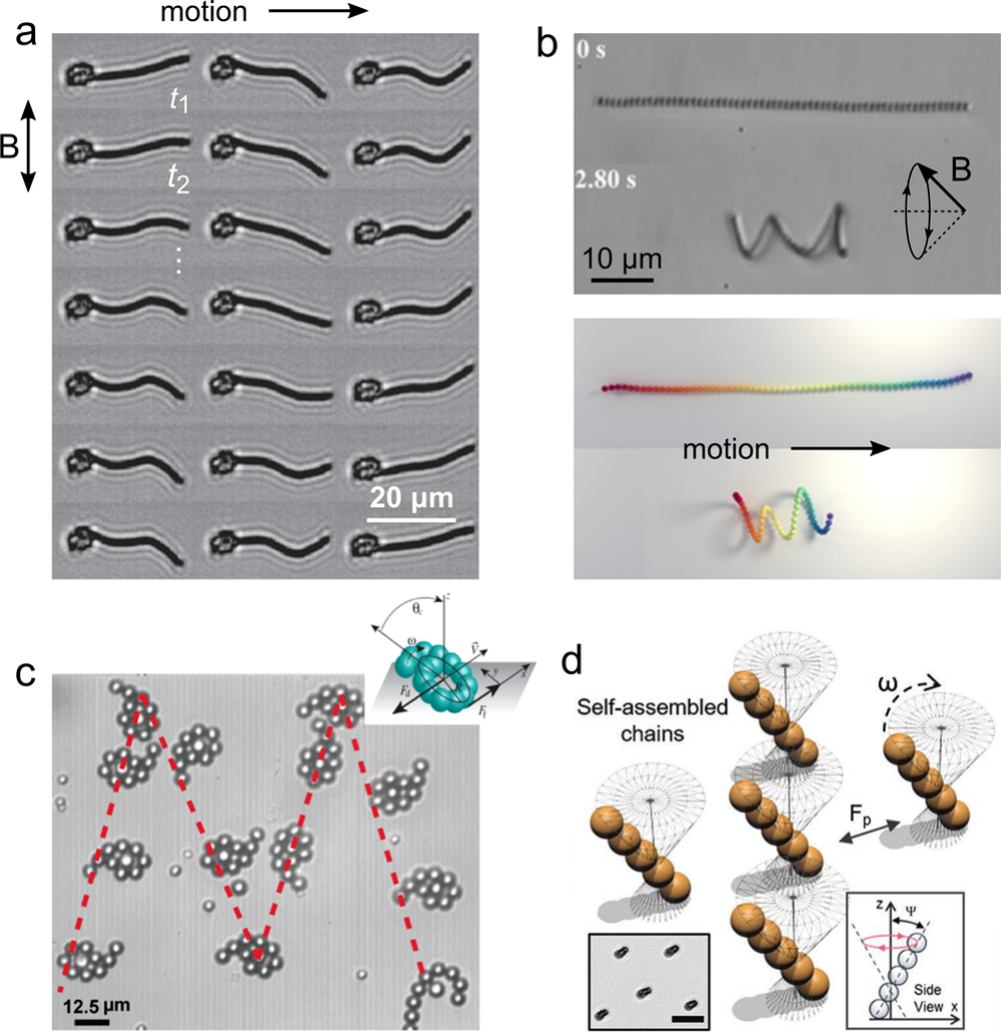
(a) A flexible chain of superparamagnetic particles attached to a red blood cell swims in an oscillating field due to a periodic sequence of nonreciprocal deformations. Reproduced with permission from ref (55). Copyright (2005) Springer Nature. (b) In a precessing field, a flexible magnetic chain coils into helix that screws through the viscous fluid as reproduced by computational models. Reproduced with permission from ref (56). Copyright (2020) National Academy of Sciences. (c) A magnetic “microlasso” in a rotating field coils around a colloidal particle, rolls the cargo along the surface, and releases it in a targeted location. Reproduced with permission from ref (59). Copyright (2017) American Chemical Society. (d) Linear chains of suparparamagnetic particles assemble in a precessing field and move along a solid wall as directed by the field.^60^ Scale bar is 25 μm. Reproduced with permission from ref (60). Copyright (2019) Wiley-VCH GmbH, Weinheim.

Linear chains of magnetic particles connected by flexible linkers enable new modes of propulsion as well as the ability to manipulate micron-scale cargo. When subject to oscillating fields, flexible magnetic chains attached to microscopic cargo exhibit traveling waves of elastic deformation that enable free swimming and cargo transport at low Reynolds numbers (Figure 6a).^55^ Without the attached cargo, chains of superparamagnetic particles break symmetry in a precessing field to adopt helical conformations that swim along the field axis (Figure 6b).^56^ More generally, particle chains flex and fold in time-varying fields to form dynamic conformations balancing magnetic, elastic, and hydrodynamic interactions among the particles.^57,58^ In particular, the formation of circular coils in rotating fields provides a basis for “microlassos” that can wrap around a micron-scale particle, transport it via surface rolling, and release it on demand by changing the driving field (Figure 6c).^59^

Even in the absence of flexible linkers, dipole–dipole interactions direct the dynamic assembly and propulsion of linear particle chains in time-varying magnetic fields.^21^ Superparamagnetic spheres moving above a planar surface assemble to form linear chains due to time-averaged dipolar interactions in an elliptically polarized, rotating magnetic field.^50^ Additional hydrodynamic interactions among the rotating particles leads these colloidal “worms” to crawl across the surface much faster than the individual particles alone. Similarly, precessing fields can induce the assembly of linear particle chains that move and interact as directed by the frequency, precession angle, and orientation of the applied field (Figure 6d).^60^ Notably, the ability to dynamically assemble and disassemble multiparticle robots on demand by changing the driving field is potentially useful for introducing (removing) them to (from) hard-to-reach places.

#### 2.3.2 2D Sheets and 3D Swarms

**Figure 7:**
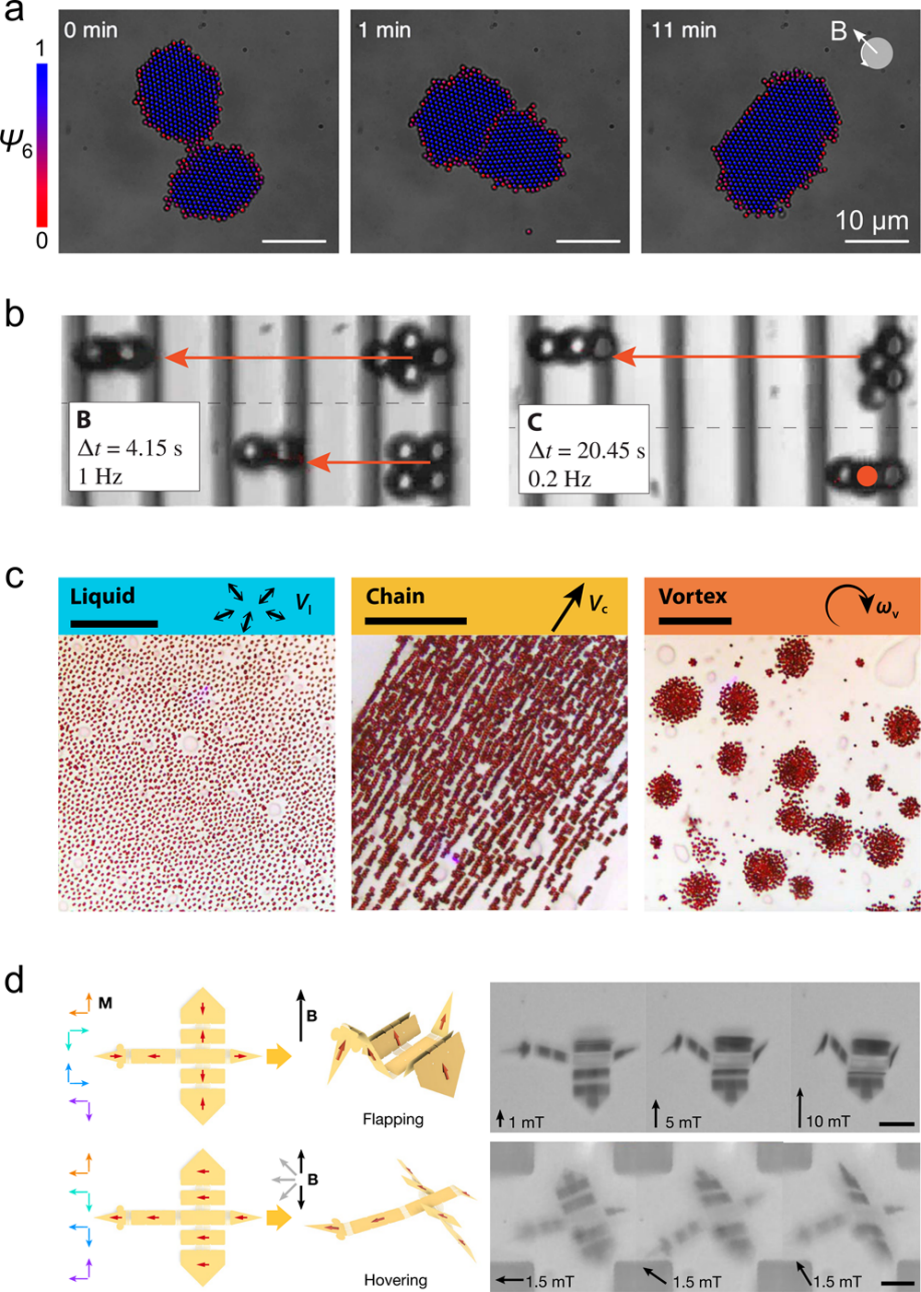
(a) Time-averaged dipolar interactions in the plane of a rotating field mediate the condensation and coalescence of superparamagnetic particle crystals.^62^ Colors denote the local orientation order parameter ψ_6_. Reproduced with permission from ref (62). Copyright (2018) American Physical Society. (b) “Microwheels” assemble in a rotating field and roll across patterned surfaces at different speeds that depend on the wheel shape and the surface topography. Wavelength of the topographic pattern is 10 μm. Reproduced with permission from ref (53). Copyright (2019) AAAS. (c) Swarms of hematite particles form dynamical phases with different functions depending on the driving field: (left) liquid phase in a oscillating field, (middle) motile chain phase in a rotating field parallel to the surface, (right) vortex phase in a rotating field perpendicular to the surface. Scale bars are 50 μm. Reproduced with permission from ref (66). Copyright (2019) AAAS. (d) A magneto-elastic sheet patterned with magnetic domains and flexible hinges folds into a microscale “bird” that “flaps” and “hovers” in the external field. Reproduced with permission from ref (68). Copyright (2019) Springer Nature.

Guided by time-averaged dipole–dipole interactions, magnetic particles assemble to form 2D crystals in the plane of a rotating field (Figure 7a).^61,62^ Once formed, these dynamic assemblies can be driven to move across planar substrates—for example, by rotating the constituent particles in an elliptically polarized, rotating field^63^ or by “rolling” the particle-assembly in a rotating field tilted out of plane.^64^ The former enables cargo transport across active colloidal “carpets”;^63^ the latter allows for the differential propulsion of various “microwheels”^64^ across patterned “microroads” (Figure 7b).^53^ In addition to dipole–dipole interactions, assemblies of field-driven particles can be held together within 3D swarms or “critters” by the hydrodynamic flows they create.^45,65^ A key advantage of these dynamic assemblies for robotic application is their reconfigurability: the same components can assemble in different ways to complete different tasks as directed by the driving field (Figure 7c).^66,67^

The assembly information^69^ encoded in the field can be augmented by additional design variables that specify the position, orientation, and connectivity of magnetic domains within flexible assemblies. The field-induced actuation of magnetic particles embedded within nonlinear elastomers enables complex changes in material shape.^70^ At the millimeter scale, these shape-changing materials have been used to create soft robots that move through diverse environments using different modes of propulsion such as undulatory swimming through liquids^71^ and rolling or walking on solid surfaces.^72^ By patterning the local magnetization and bending modulus, magneto-elastic materials can be programmed to fold spontaneously under external fields to create shape-changing origami on the micron scale (Figure 7d).^68^ Notably, the elastic component of these composite materials can be designed to respond to a variety of environmental stimuli such as temperature, osmotic pressure,^73^ and light,^74^ thereby altering the dynamics of field-driven robots. This ability to respond to local stimuli within heterogeneous environments provides a basis for “intelligent” robots that use internal feedback mechanisms^75^ to direct self-guided functions such as gradient-driven navigation.^16^ The ingredients required to create such robots are largely available; the remaining challenge is one of design. Which of the many possible driving fields, robot shapes, magnetization patterns, responsive materials, etc., should one select to create microrobots with desired capabilities? Answering this question will require advances in engineering design that leverage automation and computation to accelerate the development of self-guided microrobots.

## 3 Inverse Problem: Designing *Self-Guided* Microrobots

Mobile robots use sensors and actuators to navigate their environment and perform desired functions. Their ability to conduct the appropriate action given relevant sensory information is determined by their control system—that is, by the “brains” of the robot. For magnetic microrobots, these key elements—sensor, actuator, controller—are often external to the particle itself (Figure 2a,b).^76^ Using microscopy information (sensor), a computer algorithm (controller) alters the driving magnetic field (actuator) to achieve the desired particle response. Where feasible, these micron-scale robots based on external control systems enable useful capabilities such as drug delivery,^33^ colloidal assembly,^11,77^ cargo capture,^78^ and multimodal locomotion.^66^ However, as noted above, this external control paradigm becomes infeasible when faced with the challenges of limited information and global actuation—for example, the control of many particles with unknown positions. In this section, we consider the design of self-guided microrobots in which sensors, actuators, and controllers are embedded (often implicitly) within the particles themselves and in the driving field. Programming the behaviors of these systems can be framed as a design problem, where we seek to identify which of the many possible robot designs exhibit the desired performance. We discuss strategies for accelerating the design process using physics-based models, automated experiments, Bayesian data analysis, and machine learning approaches.

### 3.1 Framing the Design Problem

#### 3.1.1 Quantifying Performance

**Figure 8:**
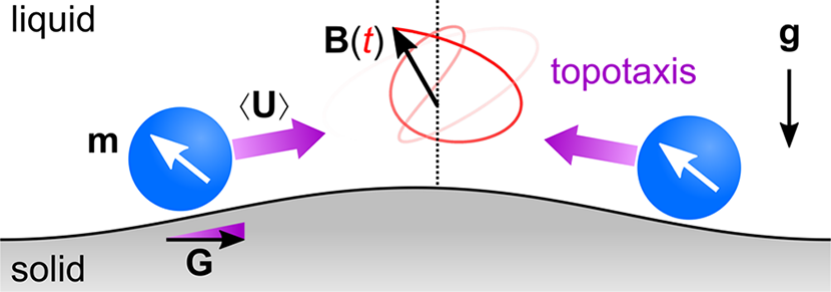
Topotaxis: ferromagnetic spheres immersed in a viscous fluid above an solid surface migrate up topographic gradients in a spatially uniform, time-varying field **B**(*t*) specifically designed for that purpose. The time-averaged particle velocity ⟨**U**⟩ is proportional to the surface gradient **G** defined relative to a symmetry axis of the driving field (dotted line). Reproduced with permission from ref (15). Copyright (2021) Royal Society of Chemistry.

The design process begins by identifying the functional capability one aims to achieve. As a specific example, we consider the design of microrobots that navigate autonomously across a solid surface in two dimensions as directed by the local surface topography—so-called topotaxis (Figure 8).^15^ Depending on the context, we may seek robots that move up, down, left, or right relative to the local surface incline with respect to the gravity direction. Importantly, we want these microrobots to be *self-guided*: particles should move “uphill”, for example, as directed by their respective environments and not by external control systems. We emphasize that topotaxis is one of many possible autonomous capabilities that could be targeted for design. Other types of gradient-driven taxis (e.g., rheotaxis,^79^ viscotaxis,^80^ chemotaxis,^16,81^ etc.) as well as conditional “if–then” operations such as cargo capture and release are discussed below.

Having identified the desired behavior, one must specify performance metrics that assess whether and to what extent that behavior is achieved in a specified context. For gradient-driven taxis, relevant performance metrics include the speed and direction of particle migration relative to the magnitude and direction of the applied gradient. In the linear response regime, the particle migration velocity ⟨**U**⟩ is linearly proportional to the gradient vector **G** as ⟨**U**⟩ = **R**·**G**, where the matrix **R** concisely summarizes the particle response. Ideally, performance metrics should be observable quantities that are readily measured in experiment. By analogy to chess programs, they should focus on desired outcomes (e.g., wins) without prescribing the path to that outcome. In topotaxis, performance is measured in terms of the particle displacement after some time (i.e., the time-averaged motion) rather than the instantaneous particle velocity, which may fluctuate in speed and direction.

#### 3.1.2 Choosing Design Variables

**Figure 9:**
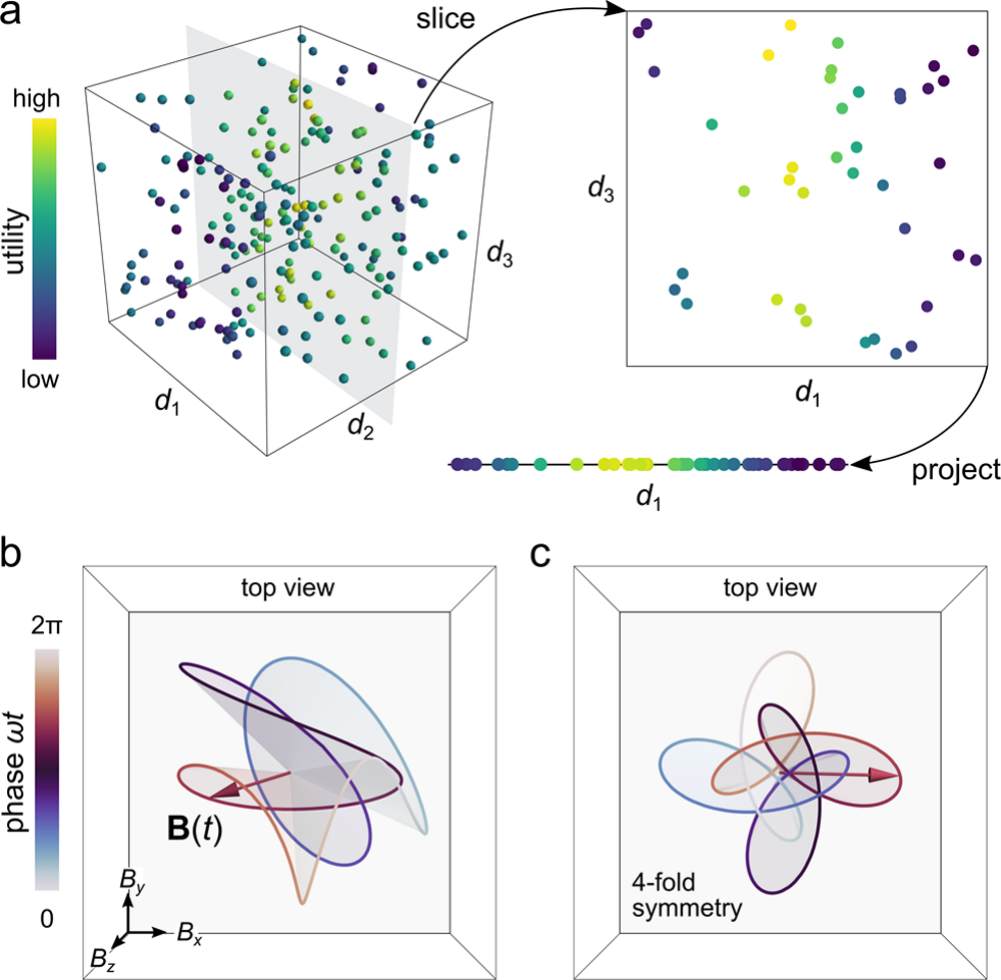
(a) A design space of three variables  is reduced to a lower-dimensional space by slicing and projecting. Each point in the original space is characterized by its utility (colored markers). Here, we fix the value of variable *d*_2_ (slice) and ignore variable *d*_3_ altogether (project). The resulting one-dimensional design space can then be explored in pursuit of “good” (high utility) designs. (b) Periodic magnetic field **B**(*t*) with frequency ω selected at random from the space of 6*N* + three-dimensional design space of Fourier components with *N* = 5 harmonics. (c) Periodic magnetic field **B**(*t*) selected at random from the design space of possible fields with *m* = 4-fold rotational symmetry about the *z*-axis.

Design variables refer to the different “knobs” that one can tune to influence robot performance. Examples include particle shape^82,83^ and composition^84^ as well as the waveform of the time-varying magnetic field. These quantities can be represented by continuous or categorical variables that together form a space of possible designs—the so-called design space (Figure 9a). As the full space of all possible designs is Vast (i.e., Very much greater than ASTronomical^22^), we make slices and projections to create a manageable space of reduced dimensionality. We fix some variables under our control (slice) and ignore others (projection). For example, we may choose a convenient particle shape to make our robot thereby excluding the possibilities afforded by alternative shapes. We may choose to ignore the time of day or the temperature of the room as these variables are thought to have negligible impact on robot performance. Together, the many ignored or uncontrolled design variables contribute to the many types of “noise” present in experimental observations.

Choosing the design space is among the most important decisions in solving a design problem—here, in programming a self-guided microrobot. Ideally, the design space should be as simple (low dimensional) as possible to facilitate exploration but sufficiently expressive to encompass nontrivial solutions. The problem of topotaxis provides an instructive example. To start, we focus our attention on a single ferromagnetic sphere moving through a viscous liquid above an inclined plane under the influence of a time-varying, spatially uniform field (Figure 8). The design space is chosen to describe the possible driving fields while fixing other details of the experiment such as particle shape. We use a truncated Fourier series to represent the time-periodic field in terms of its frequency ω and its Fourier components. In this way, a static field with *N* harmonics in three dimensions is represented by a design space with 6*N* + 3 dimensions for a specified frequency ω. Figure 9b shows one of the many possible driving fields selected at random from a design space with *N* = 5 harmonics. We conjecture that there exist some designs in this space that will drive autonomous particle migration as directed by the inclined substrate.

Symmetries in the driving field, the magnetic particle, and its environment can be helpful in reducing the design space while preventing undesired particle motions. For self-guided navigation in two dimensions, the applied field should not drive particle motion along a privileged direction when the environment is isotropic (e.g., no topotaxis on a level substrate). To prevent such field-directed motions, we can use time-periodic fields that exhibit *m*-fold rotational symmetry about an axis normal to the 2D environment. In particular, we consider fields for which a shift in phase is equivalent to a rotation in space: *R*_3_(φ_*m*_)**B**(*ωt*) = **B**(*ωt* – φ_*m*_), where *R*_3_(φ_*m*_) describes a coordinate rotation about the *z*-axis by an angle φ_*m*_ = 2π/*m* for a specified integer *m* ≤ 3. Simple examples of such fields include an oscillating field along the *z* axis and a rotating field in the *xy* plane; however, the space of possibilities becomes significantly richer with the inclusion of higher harmonics (Figure 9c shows one example for *m* = 4). Importantly, the migration of particles energized by these fields is due either to asymmetries in the particle environment (taxis) or to symmetry-breaking instabilities^52^ and not directed by the applied field. This freedom of particles to move in different directions in a common field is a defining characteristic of self-guided microrobots. Moreover, by restricting the space of possible fields to satisfy the above symmetry, we significantly reduce the number of design variables thereby facilitating the design process.

#### 3.1.3 Balancing Costs and Benefits

**Figure 10:**
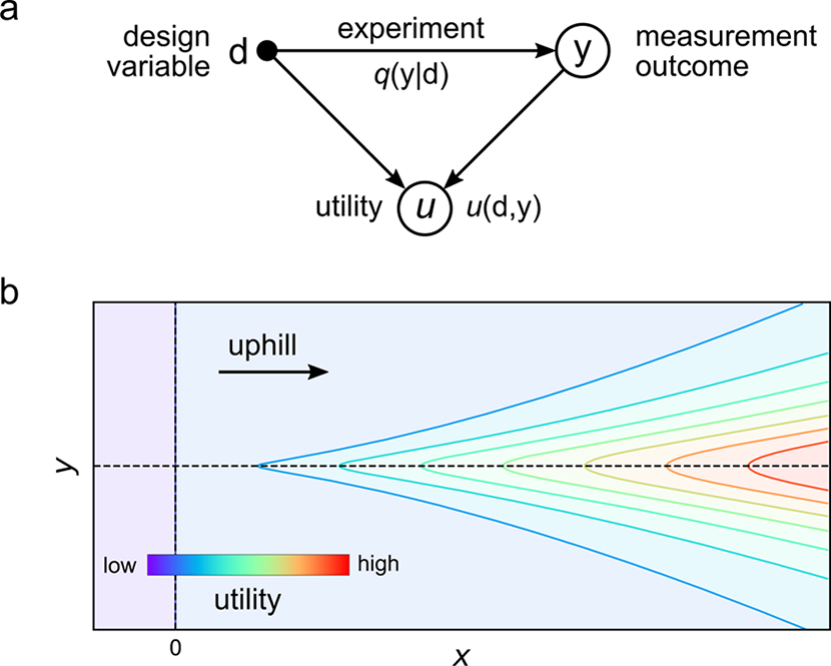
(a) Graphical representation of the design problem: The relationship between an experimental design **d** and a measurement outcome **y** is described by the conditional probability distribution *q*(**y**|**d**), which is unknown to the experimenter. The value of an experiment is described by a utility function *u*(**d**, **y**) that depends on the design (e.g., the cost of performing the experiment) and the measurement outcome (e.g., the benefit of the observed performance). We seek designs that maximize the expected utility *U*(**d**) = *∫u*(**d**, **y**)*q*(**y**|**d**)*d***y**. (b) For topotaxis on an inclined substrate, the utility function (1) favors driving fields that direct particle motion “up” the incline quickly and accurately (contours). The solid curve illustrates the particle trajectory up the incline.

Having identified a space of candidate designs, we must now quantify our preferences for some designs over others. The distinction between better and worse designs is inherently subjective and reflects the wants, priorities, and capabilities of the designer as well as the demands of the targeted application. In general, the value of a particular design can be quantified—or rather defined—by a utility function, which assigns a numeric score to each design in accordance with our preferences. Informed by performance metrics associated with each design, the utility function must balance the often conflicting demands of performance along with the costs of implementing the design (Figure10a). Continuing our example of topotaxis, good designs for the driving field propel particle motion *accurately* up the incline but also *quickly* up the incline. The utility function must weigh these competing desiderata to determine a single value for each candidate design. In our previous study, we use the following function to favor particle migration in the uphill *x*-direction
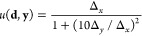
1where **d** is the design variable specifying the driving field, and **y** = [Δ_*x*_, Δ_*y*_] is the observed performance specifying the average particle displacement per cycle along directions parallel Δ_*x*_ and perpendicular Δ_*y*_ to the surface incline (Figure10b). The chosen factor of 10 sets the relative importance between the magnitude and direction of the displacement. In general, the utility can depend also on the design variable **d**—for example, when some designs require more time or resources than others.

To program the microrobot, we seek to identify one or more designs **d** from among the space of possibilities that maximize the utility function *u*(**d**, **y**) as informed by the performance metrics **y**. In this way, our initial design goal has been reformulated as an optimization problem: find the design that maximizes the utility. In practice, the search for a global optimum of a complex objective function in a high dimensional space can be a neverending challenge—particularly, when the function is costly to evaluate (e.g., requiring an experiment). For this reason, it is often wise and expeditious to lower our expectations and abandon the pursuit of “optimal” designs in favor of those “good enough” to achieve the desired capability. In the context of topotaxis, we constrain the initial design process to consider only spherical particles despite the fact that some anisotropic particles are likely to exhibit better performance (e.g., faster gradient-driven migration) in the driving field. This process of “satisficing”^85^ is all the more reasonable when we acknowledge that the design optimization problem outlined above is a quantitative fiction of our own creation. We choose the design variables, performance metrics, and utility function that quantify—albeit approximately—our goals and capabilities. If we choose wisely, this design framework can be a useful guide in accelerating the development of autonomous microrobots.

### 3.2 Accelerating the Design of Microrobots

#### 3.2.1 Generate-and-Test

Arguably the simplest algorithm for solving a design problem is the Edisonian approach of repeated trial-and-error. In this approach, we select many candidate designs spanning the space of possibilities. For each candidate, we implement the design in experiment, evaluate its performance, and quantify its utility. The process continues until an acceptable solution is found or until resources are exhausted. In its simplest form, designs are selected and evaluated independently of one another. Candidate designs may be sampled at random or selected from a regular grid in design space. The observed performance of one design does not alter the selection of other subsequent designs. As a result, the search algorithm can be conducted in parallel to accelerate the exploration of design space.

Studies by Faivre et al. on the magnetic propulsion of randomly shaped microparticles illustrate the benefits of parallel search for particle design (Figure 4c).^42,86−88^ The Authors synthesize populations of magnetic particles with irregular shapes thereby sampling a large design space of possible shapes.^86^ These designs are evaluated in parallel by observing the linear propulsion of many particles subject to a common rotating field. Using video microscopy, they identify particles that exhibit desired behaviors—for example, particles that swim the fastest^87^ or that reverse direction upon changes in the driving frequency.^42^ The three-dimensional shapes of these high performing particles are reconstructed from 2D microscopy images^42^ and copied using 3D printing to create magnetic microrobots with enhanced swimming abilities.^88^ A logical next step is the repeated iteration of particle selection and copying to enable the directed evolution of ever better designs.

Notably, the generate-and-test algorithm requires no understanding of the relationship that connects the specified design variables to the observed performance metrics. Like biological evolution, this blind search process achieves “competence without comprehension”^22,89^—for example, it reveals particle shapes that swim fast without understanding how such performance is achieved. This lack of comprehension can be an asset when the algorithm discovers surprising solutions that challenge our expectations and prior biases. For example, some randomly selected particles with irregular shapes actually swim faster than human designs based on helical or propeller-type shapes.^87^ On the other hand, our ability to understand how “good” designs achieve their performance can help to further accelerate the pace of design. Indeed, Faivre and co-workers explain their experimental observations of particle propulsion using dynamical models based on magnetic actuation and low Reynolds number hydrodynamics.^88^ As Nikolai Tesla famously criticized Edison’s trial-and-error approach, “just a little theory and calculation would have saved him 90 percent of the labour.” Like Tesla, we argue strongly for the value of models, which can anticipate experiment outcomes and guide the search through design space.

#### 3.2.2 Model-Predictive Design

**Figure 11:**
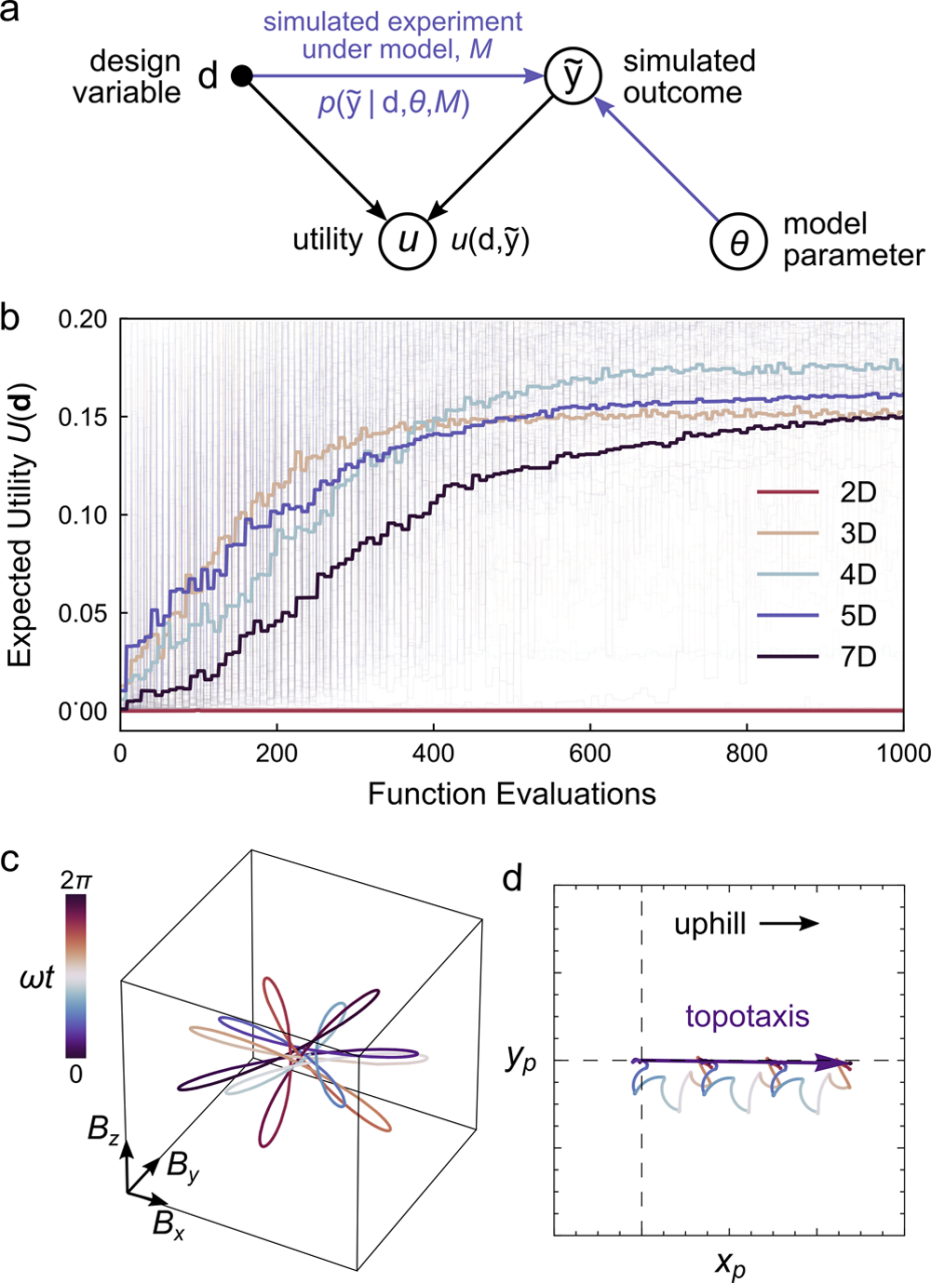
(a) Graphical representation of model predictive design. The relationship between an experimental design **d** and the outcome  of a *simulated experiment* is described by the conditional distribution  for model *M* with parameters **θ**. The value of the simulated experiment is described by a utility function . We seek designs that maximize the expected utility as predicted by the model. (b) Optimization of the expected utility *U*(**d**) using the covariance matrix adaptation evolution strategy (CMA-ES).^90^ Colors represent design spaces of varying dimensionality, reduced from the original seven-dimensional space by principal component analysis (PCA). The optimization is initialized from 50 randomly selected designs (light curves); bold curves show the average performance. (c) Time-periodic magnetic field **B**(*t*) with 6-fold rotational symmetry designed to drive topotactic particle migration up inclined surfaces. (d) Simulated *xy* trajectory of a ferromagnetic sphere driven by the field in (c). Reproduced with permission from ref (15). Copyright (2021) Royal Society of Chemistry.

Generative models that predict the outcomes of imagined experiments can be used in place of experimental data to guide the design process (Figure 11a). Such models take as input the specified design variables and return as output the predicted performance metrics that enable the evaluation of candidate designs. To be useful for this purpose, models should provide reasonably accurate predictions of experimental outcomes at lower “costs” than the experiments themselves (e.g., in terms of time and other resources). As an illustrative example, we return to the design of time-varying fields for directing the topotactic migration of ferromagnetic spheres.^15^ Using dynamical models of particle motion, we can predict *in silico* how the particle will move along an inclined surface in a specified driving field. Importantly, such simulations can be performed in a small fraction of the time required to conduct an analogous experiment. It is therefore possible to evaluate millions of candidate designs for the driving field and select the most promising from the design space of rotationally symmetric fields.^15^ This approach is identical with the generate-and-test algorithm of the preceding section applied now to model predictions rather than experimental observations.

When possible, model-predictive design based on accurate and efficient models can identify solutions more quickly and with deeper understanding than experimental trial-and-error alone. Physics-based models of particle dynamics provide idealized descriptions that make explicit assumptions about which features of the system are important (e.g., magnetic moment, fluid viscosity) and which are not (e.g., inertial effects, phase of the moon). Models of topotaxis may neglect the real effects of Brownian motion and surface roughness on particle dynamics as well as those due to hydrodynamic and magnetic interactions between neighboring particles. Nevertheless, these idealized models make useful albeit approximate predictions that guide the search for effective driving fields as evidenced by subsequent experiments.^15^

In contrast to experimental measurements that provide uncertain and incomplete information, model-based simulations provide a comprehensive description of particle dynamics in three dimensions, which can be analyzed to deepen our understanding. From models of topotaxis, we learn that the speed of particle migration up an inclined substrate grows as the square of the driving frequency, reaching its maximal value when the particle’s moment can just keep pace with the changing magnetic field. Such insights from the model can be used to tailor the design space and direct further improvements in microrobot performance. In particular, simulation results can be used to reduce the dimensionality of the design space thereby facilitating its exploration in experiment. Using simulated data for topotaxis, we showed that principle component analysis (PCA) can reduce the design space for the driving field from seven to three dimensions without significantly reducing design performance.^15^ The resulting three-dimensional space can now be explored using automated experiments to identify high performing designs that account for additional physics neglected by the model (Figure 11b–d).

Of course, model-predictive design is not always possible when models are uncertain, inaccurate, expensive, and/or absent altogether. In the sections that follow, we address these different scenarios in turn. Simulations of system performance often depends on model parameters (e.g., magnetic moment, surface separation) that are uncertain or unknown thereby limiting the precision of model predictions. Methods of Bayesian data analysis provide a principled approach for learning model parameters from experimental data and quantifying the uncertainty of model predictions (Section 3.2.3). When physics-based models are absent or infeasible, the design process can benefit from machine learning approaches based on surrogate models informed by experimental data (Section 3.2.4). Ultimately, the best approach for any given problem is a thoughtful mixture of all-of-the-above that respects known physics, incorporates available data, and enables efficient computation.

#### 3.2.3 Design under Uncertainty

In the context of magnetic microrobots, we are fortunate to have strong physics-based models with which to guide the design process. Such models involve physical parameters such as the particle radius and the fluid viscosity that must be specified to predict the particle dynamics. When these parameters are unknown or uncertain, they must be estimated from experimental data. Bayesian inference^91−93^ uses probability theory to describe our uncertain knowledge of model parameters **θ** and provides a normative framework for changing our beliefs about their likely values in response to observed data **y**. More explicitly, Bayes’ theorem describes how the prior distribution *p*(**θ**|*M*) for the parameters is updated to obtain the posterior distribution *p*(**θ**|**y**, *M*) conditioned on the data
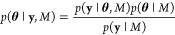
2Here, the likelihood function *p*(**y**|**θ**, *M*) provides a probabilistic description of the observed data that accounts for stochastic variation (i.e., “noise”) due to thermal fluctuations, particle dispersity, heterogeneous environments, and measurement error among other possible sources. For parameter estimation, the so-called evidence *p*(**y**|*M*) in the denominator is simply a normalizing constant for the posterior distribution. Importantly, all of these distributions are conditioned on the assumption that the observed data **y** are generated by the proposed model *M*. As discussed below, it is essential to challenge this assumption and confirm the (approximate) validity of the model at each stage of the design process.

Estimating model parameters using Bayes’ theorem 2 is often easy in principle but challenging in practice. For all but the simplest models, the posterior distribution cannot be calculated analytically and instead requires numerical methods of probabilistic programming. For a small number of model parameters (typically, ≤4), the posterior can be computed on a discrete grid of candidate parameter values and interpolated to approximate expectations of the form
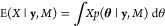
3where *X* is some quantity of interest (e.g., a model parameter or prediction). With additional parameters, sampling methods such as Markov Chain Monte Carlo (MCMC) can be used to generate random parameter samples from the posterior distribution and compute approximate expectations. There exist a growing number of probabilistic programming tools (e.g., Stan,^94^ PyMC3,^95^ Julia^96^) that provide efficient implementations of these sampling techniques. Alternatively, one can seek to approximate the posterior in terms of simpler distributions (e.g., the multivariate normal) using the Laplace approximation^91^ or variational inference.^97^

**Figure 12:**
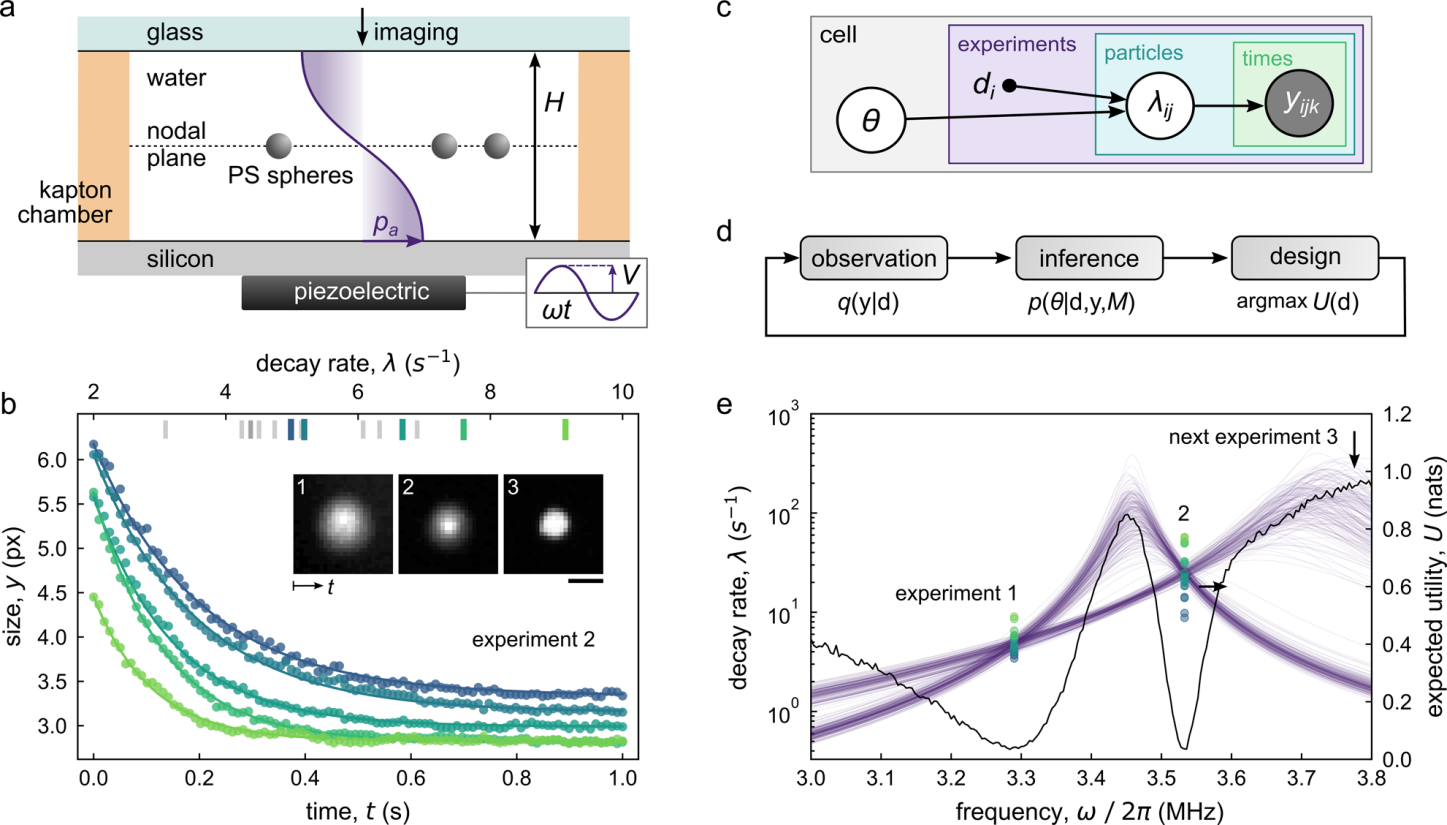
Bayesian inference and design for quantifying acoustic particle levitation.^98^ (a) Experimental schematic of a resonant acoustic cell containing polystyrene tracer spheres. (b) Application of an acoustic field causes the spheres to levitate to the nodal plane as observed by optical microscopy. Scale bar is 15 μm. (c) Graphical representation of the hierarchical model. The observed size *y*_*ijk*_ at time point *t*_*i*_ of particle *j* in experiment *i* depends on particle-level parameters λ_*ij*_ (e.g., the local acoustic field), the experiment design **d**_*i*_ (e.g., the applied frequency), as well as cell-level parameters **θ** (e.g., the resonant frequency). (d) Cell-level parameters **θ** can be learned using a minimal number of experiments through an iterative cycle of observation, inference, and design. (e) After two experiments (solid markers), the predicted dependence of the decay rate λ on the applied frequency ω (purple curves, left *y*-axis) shows two hypotheses: the resonant frequency of the cell is ca. 3.45 or 3.7 MHz. The next experiment 3 is chosen to maximize the expected utility (black curve, right *y*-axis) and thereby discriminate between these competing interpretations of the data. This iterative process converges to accurate estimates of the cell-level parameters using few automated experiments. Reproduced with permission from ref (98). Copyright (2021) Royal Society of Chemistry.

The Bayesian paradigm is particularly useful in analyzing tracking data for multiple particles that exhibit different sources of stochastic variation. In the context of topotaxis, the analysis of particles moving on topographic landscapes may require one to consider differences among the particles (e.g., their magnetic moments and heights above the substrate), differences in their environments (e.g., the direction and magnitude of the local inline), effects of Brownian motion, and measurement error in particle tracking. These many layers of uncertainty that contribute to the observed data can be described using Bayesian hierarchical models. Figure 12 provides an illustrative example of this modeling framework as applied to the acoustic levitation and propulsion of particles in a standing acoustic field.^98^ This example includes two levels of noise: one due to the heterogeneity of the acoustic field (i.e., the sound wave is louder here and softer there), and another due to measurement error in the particle response. Bayesian analysis of this hierarchical model enables the accurate characterization of the heterogeneous acoustic field using noisy measurements of many particles. Moreover, by modeling the particle population, we improve the precision of parameter estimates for each individual particle.

Generative models required for Bayesian inference enable posterior predictions of simulated data  conditioned on observed data **y**
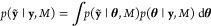
4Importantly, these predictions are only as accurate as the model  and as precise as the parameter estimates *p*(**θ**|**y**, *M*). It is therefore necessary to criticize the fitted model by assessing its ability to describe the observed data and to predict future data yet to be observed. The process of Bayesian model criticism asks the basic question: does simulated data from the fitted model “look like” the observed data from experiment? If we cannot distinguish the observed data from an ensemble of simulated data, then the model should be provisionally accepted.^99,100^ Alternatively, when model predictions differ systematically from the observed data, we must decide whether to reject the model and seek better alternatives or to proceed cautiously with greater appreciation of the model’s limits. Such posterior predictive checks (PPCs) can be quantified using the formalism of hypothesis testing, where the null hypothesis states that the observed data is generated by the fitted model.

Given a fitted model that passes our PPCs and faithfully describes the observed data, we can now put it to use in guiding the design process. In particular, we seek the design that maximizes the expected utility as predicted by our probabilistic model conditioned on the observed data **y**
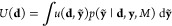
5Here, the utility function  depends on the design **d** and the predicted outcome  of a future experiment as described by the posterior predictive distribution . Importantly, this process of observation, inference, and design can be iterated to guide the execution of successive experiments and improve design performance.^101^ Recently, we demonstrated this Bayesian design approach to quantify the levitation and propulsion of colloidal particles in acoustic fields.^98^ In that work, the utility function was chosen to learn model parameters using the fewest number of experiments (i.e., to maximize the information provided by each experiment) by tuning design variables such as the frequency and magnitude of the applied field. By using a different utility function, the same approach can guide the design processes to the desired performance—for example, to maximize the expected intensity of the resonant acoustic field. Whether for knowledge or performance, the Bayesian design framework provides a principled approach for navigating uncertainty and incorporating new data in pursuit of the design objective(s).

#### 3.2.4 Machine Learning with Surrogate Models

When physics-based models are unavailable or prohibitively expensive to evaluate, we can substitute heuristic models trained on experimental data to describe the relationship between design variables and the observed quantities of interest. In the Bayesian paradigm, such surrogate models provide a probabilistic description of the observed data conditioned on the model parameters.^102^ In this sense, they are no different from physics-based models. For example, we might adopt a surrogate model in which the observed outputs depend linearly on the specified inputs with additive Gaussian noise. Given experimental data, we can train the model to learn unknown parameters (i.e., the linear coefficients relating inputs to outputs) and make probabilistic predictions using the methods of Bayesian inference outlined above. Owing to the simplicity of the model, this inference problem can be solved analytically using linear algebra. The result—known as linear regression—is one of many methods of probabilistic machine learning that seek to learn predictive relationships from available data.

**Figure 13:**
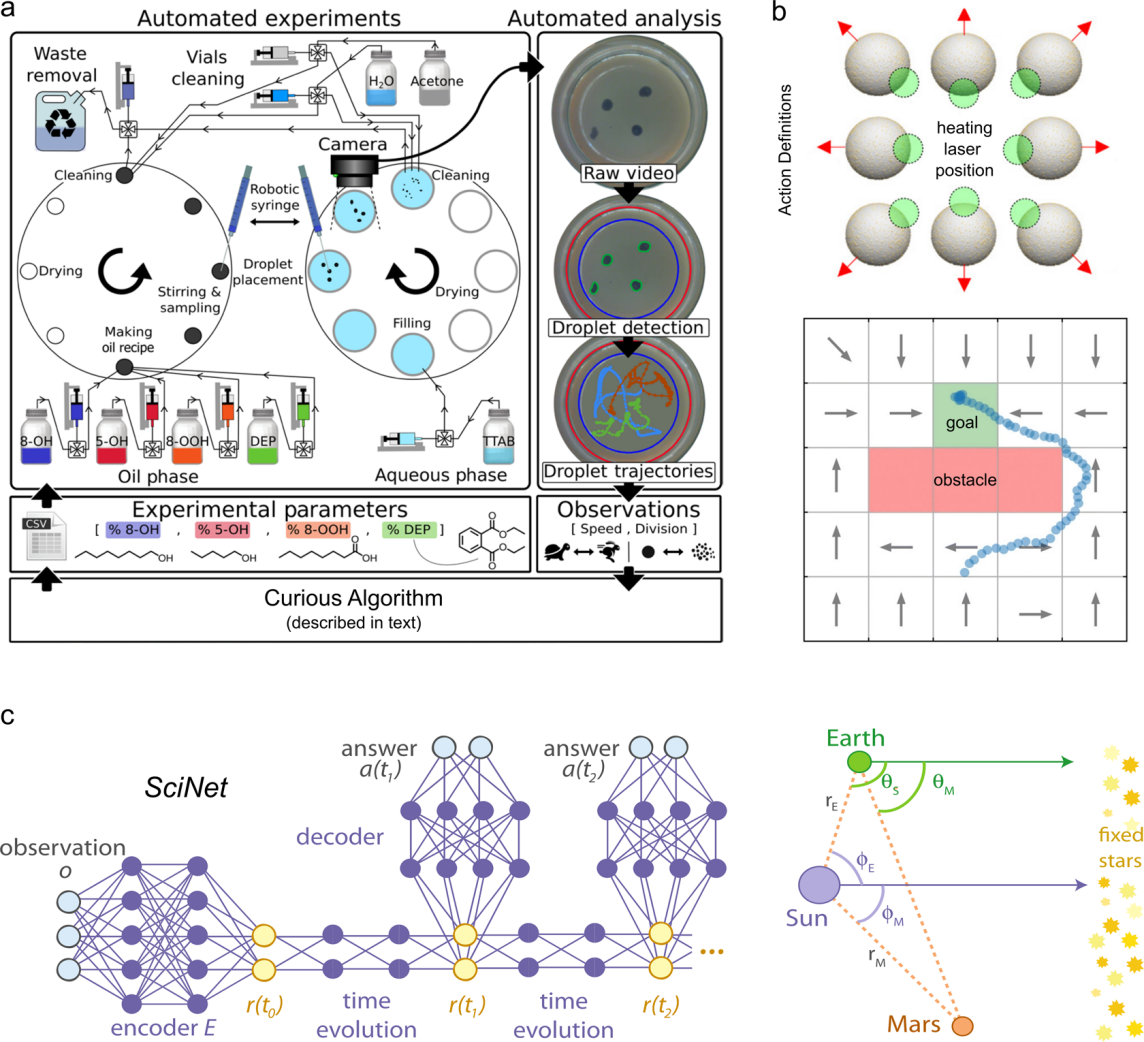
(a) An automated platform explores the self-propulsion and division of oil droplets in water.^103^ Within a series of automated experiments, the design variables (i.e., the drop composition) are selected by a “curious algorithm” that seeks to sample uniformly the space of observed behaviors—namely, the drop speed and division count. Reproduced with permission from ref (103). Copyright (2020) AAAS. (b) Automated experiments based on reinforcement learning identify optimal policies that guide thermophoretic propulsion of a 2 μm particle around obstacles (red) to a specified goal (green). Reproduced with permission from ref (104). Copyright (2021) AAAS. (c) The *SciNet* model uses an encoder–decoder architecture to identify concise representations of physical systems. Using time series data on the positions of the Sun and Mars viewed from Earth (θ_*S*_, θ_*M*_), the model learns a new heliocentric representation based on the angles ϕ_*E*_ and ϕ_*M*_. Reproduced with permission from ref (105). Copyright (2020) American Physical Society.

The surrogate models of machine learning differ from physics-based models in regard to their interpretation, extrapolation, and computation. Physics-based models are typically constructed from components that exist independently of the system as a whole. The magnetic moment that appears in a model of topotaxis is—approximately—the same magnetic moment one would infer for the same particle in a different context. By contrast, the parameters of surrogate models cannot—in general—be interpreted as meaningful quantities independent of the model as a whole. Moreover, the predictions of surrogate models are most accurate on the domain of the training data and can produce nonsensical results when applied outside of that domain. In short, these models are good at interpolation but bad at extrapolation. Accurate physics-based models can extrapolate beyond the domain of observed data to make useful predictions in unfamiliar contexts. Unfortunately, the computational cost of such models can become prohibitively expensive as systems grow in complexity. By contrast, a major focus of machine learning is the development of general-purpose models and algorithms (e.g., neural networks and backpropagation) that enable efficient learning and prediction based on large data sets. The following examples help to illustrate how these machine learning approaches can be used to guide experimental design (Figure 13).

Cronin et al. used a “curious” algorithm to guide the discovery of oil-in-water droplets that exhibit life-like behaviors such as self-propulsion and division (Figure 13a).^103^ By varying the composition of the oil phase (the design variable), droplets are observed to swim at different speeds and divide more or fewer times (the performance metrics). As the mechanisms of drop propulsion and division are uncertain, the relationship between the design variables and the performance metrics was approximated by a simple linear model. Guided by the model, the curious algorithm seeks to choose a sequence of designs that sample uniformly the space of observed performance metrics. In contrast to methods of exploration that sample random designs, the curious algorithm explores the space of possible behaviors to avoid oversampling degenerate designs that produce similar results. Using their fully automated “dropfactory”, the authors perform ∼6000 experiments to reveal the bounds of achievable performance and the diversity of drop behaviors. The experimental results discover patterns of behavior that stimulate hypotheses about drop propulsion and division by which to guide further understanding and design.

Muiños-Landin et al. apply model-free reinforcement learning to identify actuation policies that direct the thermophoretic propulsion of a Brownian microparticle to a targeted location (Figure 13b).^104^ Each candidate policy specifies the propulsion direction from eight possibilities for each of the 5 × 5 coarse-grained particle locations. To identify the optimal design from these 200 possibilities, a series of automated experiments is conducted to iteratively improve the initial policy using an algorithmic reward system. After ∼7 h of learning by repeated trial and error, the system converges to an optimal policy that allows the particle to navigate obstacles and reach the targeted location despite the confounding effects of Brownian motion. Notably, this type of reinforcement learning does not rely on generative models of the particle dynamics but rather repeated experience to achieve the desired performance. Subsequent analysis of the learned policies can offer useful insights into the underlying physics and the “free-floating rationale”^22^ that enable the system’s performance. The authors suggest the next step is a more robust policy, one that does not rely on global position as states, and instead uses local sensing.

Ultimately, we would like machine-based learning algorithms that discover “real patterns”^106^ hidden in experimental data and explain these patterns in terms of physical laws.^107^ There has been significant progress in the algorithmic extraction of dynamical equations from experimental data^108^—for example, learning Newton’s laws from the chaotic dynamics of a double pendulum.^109^ These approaches make assumptions about the representation of the system dynamics in terms of state variables governed by differential equations. Recently, Iten and co-workers^105^ used a different approach—a neural network architecture modeled on the human reasoning process—to directly learn efficient representations of experimental data without such prior assumptions (Figure 13c). In their model (*SciNet*), experimental observations are first compressed into a simpler representation (encoding), which is then used to answer questions or make predictions about the system (decoding). The Authors demonstrate the capabilities of this approach using toy problems from different areas of physics. The *SciNet* model learns the relevant parameters of a damped pendulum (i.e., the frequency and damping factor) as well as conservation laws governing particle collisions. Based on time series data for the angular position of the Sun and Mars viewed from Earth, the *SciNet* model learns a heliocentric representation with which to efficiently describe planetary dynamics of the solar system (Figure 13c, right). While the extension of these methods to problems of increasing complexity remains to be demonstrated, the ability to interpret and understand efficient representations learned from experimental data has the potential to accelerate the design of self-guided microrobots among other physical systems.

## 4 Outlook

Guided by the example of topotactic rollers, we can envision many related opportunities for self-guided microrobots that sense and respond to their local environment. The dynamics of rigid particles in viscous fluids is sensitive to variations in the fluid velocity, viscosity, and the proximity of solid boundaries. With suitable design, such environmental cues can be used to direct the self-guided motions of magnetic particles in time-varying fields. Interesting design targets include microrobots that swim up viscosity gradients (viscotaxis^80^), against fluid flows (rheotaxis^79^), or toward solid boundaries. Together these capabilities would enable self-guided robots that can navigate microfluidic networks such as the human vascular system. Additional sensing capabilities can be introduced using responsive particles that modulate their size, shape, or elasticity in response to local stimuli such as pH or temperature.

Beyond gradient driven taxis, self-guided microbots could be designed to exhibit conditional “if–then” responses whereby different environments trigger different dynamical behaviors. For example, a pH-responsive microbot might swim toward solid boundaries in acidic conditions and away from such boundaries in basic conditions due to pH-dependent changes in particle shape. With each additional behavior, the design challenge grows, likely requiring more design variables tuned to greater precision. Navigating this growing space of possible designs will benefit from modularity whereby complex behaviors are decomposed into simpler, independent components.

The pursuit of microrobots with increasing physical intelligence will further benefit from particles with memory^110^ whose behavior is conditioned on internal states as well as the local environment. For example, a primitive microrobot designed for capturing cargo might exhibit different dynamics conditioned on two states: “empty” and “full”. In this way, it may be possible to design self-guided microrobots that swim “upstream” when “empty” in search of cargo and “downstream” when “full” to return home. Such speculation raises important questions about the limits of encoding complex behaviors in the current space of possible designs. Which behaviors are possible? Which require the affordance of new design variables?

Ultimately, the creation of microrobots that mimic—even primitively—the autonomous capabilities of living cells requires advances in the design of material systems that convert input signals from the environment into output actions to achieve desired functions.^16^ Historically, robotic systems have relied on sensors, actuators, and controllers based on digital electronics. This paradigm will continue to contribute to the development of colloidal robots operating in structured fluid environments.^8,111^ However, there is growing interest in a complementary perspective in which robotic functionality is embedded within the constituent materials themselves—so-called physical intelligence.^14^ Such material systems function as analog computers that map input signals to output responses by way of their nonlinear physicochemical dynamics. Programming the desired input–output relationships is achieved through the design of these systems and their dynamics. This design problem—like that of many material systems—is challenging because the dimensionality of the design space is large, and the models used to predict performance are imperfect and uncertain.

Despite these challenges, the realization of self-guided microrobots with programmable functions—particularly those powered and directed by magnetic fields—appears close at hand. Using available materials and fabrication strategies, the design space of possible robots with prescribed shape, magnetization, composition, elasticity, and stimuli response has become sufficiently expressive as to encompass (most likely) a variety of primitive functions conditioned on the local environment. By expanding the design space to include complex time-varying fields, even simple magnetic particles are capable of autonomous navigation directed by topographic landscapes^15^ and other gradients. It remains to determine which of the many possible designs will achieve the desired functions. The pace of the design process will continue to accelerate by leveraging advances in experiment automation, model computation, statistical inference, and machine learning. Like Geppetto wishing his puppet Pinocchio to life, we are optimistic that the magnetic “marionettes” of today will soon be freed from their external controllers to enable new opportunities for autonomous microrobots in material science, environmental sustainability, and biomedicine.

## Supporting Information Available

(1) A brief review of magnetic multipoles, (2) basic physics of magnetic actuation, (3) analysis of two ferromagnetic spheres in a rotating magnetic field, and (4) analysis of ferromagnetic and superparamagnetic rollers (PDF)
