# Supplementary material for: Accelerating the Design of Self-Guided Microrobots in Time-Varying Magnetic Fields
Source: JACS Au. 2023 Mar 10;3(3):611–27. doi: 10.1021/jacsau.2c00499 (PMC10052236; doi:10.1021/jacsau.2c00499)
Supplement: Supplementary file 1 — au2c00499_si_001.pdf [file au2c00499_si_001.pdf]

Supporting information for: Accelerating the design of self-guided microrobots in time-varying magnetic fields

Kiran Dhatt-Gauthier, Dimitri Livitz, Yiyang Wu, and Kyle J. M. Bishop *

Department of Chemical Engineering, Columbia University, New York, NY, USA

E-mail: kyle.bishop@columbia.edu

Contents

1 Magnetic Multipoles **S3**

Dipole Moment, **m** S3

Quadrupole Moment, **Q** S4

2 Basic Physics of Magnetic Actuation **S7**

2.1 Magnetic Micro-Particles S7

2.2 Magnetic Forces and Torques S10

3 Two Ferromagnetic Spheres in a Rotating Field **S13**

Magnetic Force and Torque S13

Hydrodynamics S14

Stability of Rigid-Body Rotation S14

4 Magnetic Rollers in a Rotating Field **S17**

4.1 Ferromagnetic Rollers in a Rotating Field S17

4.2 Superparamagnetic Rollers in a Rotating Field S18

References **S20**

1 Magnetic Multipoles

The magnetostatic field $\mathbf{H}(\mathbf{r})$ is governed by

$$\nabla \cdot \mathbf{H} = \rho_{\mathrm{m}} \quad \text{and} \quad \nabla \times \mathbf{H} = 0 \tag{1}$$

where $\rho_{\mathrm{m}}(\mathbf{r}) = -\nabla \cdot \mathbf{M}(\mathbf{r})$ is the density of magnetic "charges" by analogy to electrostatics.[S1] We consider a "particle" as some finite region of space enclosed by a surface S_{p}, outside of which the magnetization $\mathbf{M}$ and the charge density ρ_{m} are zero. Because the curl of the field is zero, we can write it as the gradient of a scalar potential, $\mathbf{H} = -\nabla\varphi$, where the potential φ is governed by the Poisson equation

$$\nabla^2 \varphi = -\rho_{\mathrm{m}} \tag{2}$$

Outside of the particle, the disturbance potential due to the particle can be expanded as

$$\varphi(\mathbf{r}) = \frac{1}{4\pi}\left(\frac{q}{r} + \frac{\mathbf{r}\cdot\mathbf{m}}{r^3} + \frac{\mathbf{rr}:\mathbf{Q}}{2r^5} + \dots\right) \tag{3}$$

where $q = 0$ is the (non-existant) magnetic monopole moment, $\mathbf{m}$ is the dipole moment, and $\mathbf{Q}$ is the quadrupole moment.[S2]

Dipole Moment, m

The dipole moment is defined by the following integral over the particle volume[S2]

$$\mathbf{m} = \int_{V_{\mathrm{p}}} \mathbf{r}\rho_{\mathrm{m}}(\mathbf{r})dV \tag{4}$$

Using the Poisson equation (2) and Green's second identity, the dipole moment can be expressed by the following integral over the particle surface

$$\mathbf{m} = \int_{S_\mathrm{p}} (\mathbf{n}\varphi - \mathbf{r}(\mathbf{n} \cdot \nabla\varphi))dS \tag{5}$$

where $\mathbf{n}$ is the unit normal vector directed out from the surface. Importantly, this integral can be evaluated over any surface that encloses the particle; it is convenient to choose a spherical surface centered on the particle origin ($r = 0$). Substituting equation (3) for the potential and evaluating the integral, one can confirm that the dipole moment $\mathbf{m}$ of the multipole expansion is indeed the same moment defined by equation (4).

Alternatively, the dipole moment can be viewed as two point charges $\pm q$ separated by a displacement $\mathbf{d}$.[S3] The corresponding potential is

$$\varphi(\mathbf{r}) = \frac{1}{4\pi}\left(\frac{q}{|\mathbf{r} - \frac{1}{2}\mathbf{d}|} - \frac{q}{|\mathbf{r} + \frac{1}{2}\mathbf{d}|}\right) = \frac{\mathbf{r} \cdot (q\mathbf{d})}{4\pi r^3} + O(d^2) \tag{6}$$

where $\mathbf{m} = q\mathbf{d}$ is identified as the dipole moment. This perspective is useful in evaluating the force and torque on the dipole as

$$\mathbf{F} = q\mathbf{B}(\tfrac{1}{2}\mathbf{d}) - q\mathbf{B}(-\tfrac{1}{2}\mathbf{d}) = q\mathbf{d} \cdot \nabla\mathbf{B}(\mathbf{0}) + O(d^2) \tag{7}$$

$$\mathbf{T} = \frac{1}{2}\mathbf{d} \times q\mathbf{B}(\tfrac{1}{2}\mathbf{d}) + \frac{1}{2}\mathbf{d} \times q\mathbf{B}(-\tfrac{1}{2}\mathbf{d}) = q\mathbf{d} \times \mathbf{B}(\mathbf{0}) + O(d^2) \tag{8}$$

where $\mathbf{B} = \mu_0\mathbf{H}$ is the magnetic B-field outside of the particle. The same result is obtained by integrating the Maxwell stress tensor over the particle surface.

Quadrupole Moment, Q

The quadrupole moment is defined as

$$\mathbf{Q} = \int_{V_\mathrm{p}} (3\mathbf{r}\mathbf{r} - r^2\boldsymbol{\delta})\rho_\mathrm{m}(\mathbf{r})dV \tag{9}$$

such that $\mathbf{Q}$ is symmetric ($Q_{ij} = Q_{ji}$) with zero trace ($\sum_i Q_{ii} = 0$).[S2] Using the Poisson equation (2) and Green's second identity, the quadrupole moment can be expressed by the following integral over the particle surface

$$\mathbf{Q} = \int_{S_\mathrm{p}} \big(2(3\mathbf{nr} - r\boldsymbol{\delta})\varphi - (3\mathbf{rr} - r^2\boldsymbol{\delta})(\mathbf{n}\cdot\nabla\varphi)\big)dS \tag{10}$$

Substituting equation (3) for the potential and evaluating the integral, one can confirm that the quadrupole $\mathbf{Q}$ of the multipole expansion is the same moment defined by equation (9).

Alternatively, the quadrupole moment can be viewed as two dipoles $\pm q\mathbf{d}$ separated by a displacement $\mathbf{d}'$.[S3] The corresponding potential is

$$\begin{aligned}\varphi(\mathbf{r}) &= \frac{1}{4\pi}\left(\frac{(\mathbf{r}-\frac{1}{2}\mathbf{d}')\cdot(q\mathbf{d})}{|\mathbf{r}-\frac{1}{2}\mathbf{d}'|^3} - \frac{(\mathbf{r}+\frac{1}{2}\mathbf{d}')\cdot(q\mathbf{d})}{|\mathbf{r}+\frac{1}{2}\mathbf{d}'|^3} + O(d^2)\right)\\ &= \frac{1}{4\pi}\left(\frac{3\mathbf{rr}}{r^5} - \frac{\boldsymbol{\delta}}{r^3}\right) : (q\mathbf{dd}') + O(d^2, d'^2)\\ &= \frac{1}{4\pi}\frac{q\big(3(\mathbf{dd}'+\mathbf{d}'\mathbf{d}) - 2(\mathbf{d}\cdot\mathbf{d}')\boldsymbol{\delta}\big) : \mathbf{rr}}{2r^5} + O(d^2, d'^2)\end{aligned} \tag{11}$$

By comparison with equation (3), the quantity $\mathbf{Q} = q\big(3(\mathbf{dd}'+\mathbf{d}'\mathbf{d}) - 2(\mathbf{d}\cdot\mathbf{d}')\boldsymbol{\delta}\big)$ is identified as the symmetric, traceless quadrupole moment. The force on the quadrupole is

$$\begin{aligned}\mathbf{F} &= q\mathbf{B}(\tfrac{1}{2}(\mathbf{d}+\mathbf{d}')) - q\mathbf{B}(\tfrac{1}{2}(-\mathbf{d}+\mathbf{d}')) - q\mathbf{B}(\tfrac{1}{2}(\mathbf{d}-\mathbf{d}')) + q\mathbf{B}(\tfrac{1}{2}(-\mathbf{d}-\mathbf{d}'))\\ &= \tfrac{1}{2}q(\mathbf{dd}'+\mathbf{d}'\mathbf{d}) : \nabla\nabla\mathbf{B}(\mathbf{0}) + O(d^2, d'^2)\end{aligned} \tag{12}$$

Noting that the Laplacian of the field is zero ($\nabla^2\mathbf{B} = 0$), the force can be expressed in terms of the quadrupole $\mathbf{Q}$ as

$$\mathbf{F} = \tfrac{1}{6}\mathbf{Q} : \nabla\nabla\mathbf{B}(\mathbf{0}) \tag{13}$$

The torque on the quadrupole is given by

$$\begin{aligned}\mathbf{T} &= \tfrac{1}{2}(\mathbf{d}+\mathbf{d}') \times q\mathbf{B}(\tfrac{1}{2}(\mathbf{d}+\mathbf{d}')) - \tfrac{1}{2}(-\mathbf{d}+\mathbf{d}') \times q\mathbf{B}(-\tfrac{1}{2}(\mathbf{d}+\mathbf{d}')) \\ &\quad - \tfrac{1}{2}(\mathbf{d}-\mathbf{d}') \times q\mathbf{B}(\tfrac{1}{2}(\mathbf{d}-\mathbf{d}')) + \tfrac{1}{2}(-\mathbf{d}-\mathbf{d}') \times q\mathbf{B}(\tfrac{1}{2}(-\mathbf{d}-\mathbf{d}')) \\ &= q\mathbf{d} \times (\mathbf{d}' \cdot \nabla\mathbf{B}(\mathbf{0})) + q\mathbf{d}' \times (\mathbf{d} \cdot \nabla\mathbf{B}(\mathbf{0})) + O(d^2, d'^2) \\ &= \big(q(\mathbf{d}\mathbf{d}' + \mathbf{d}'\mathbf{d}) \cdot \nabla\big) \times \mathbf{B}(\mathbf{0}) + O(d^2, d'^2)\end{aligned} \tag{14}$$

Noting that the curl of the field is zero ($\nabla \times \mathbf{B} = 0$), this expression simplifies as

$$\mathbf{T} = \tfrac{1}{3}(\mathbf{Q} \cdot \nabla) \times \mathbf{B}(\mathbf{0}) \tag{15}$$

or, equivalently, in index notation $T_i = \frac{1}{3}\epsilon_{ijk} Q_{jl} \partial_{r_l} B_k$.

2 Basic Physics of Magnetic Actuation

2.1 Magnetic Micro-Particles

A magnetic particle[S1,S4] creates a dipolar disturbance field $\mathbf{H}_\mathrm{d}$ characterized by the particle's magnetic moment $\mathbf{m}$ (Fig. S1a)

$$\mathbf{H}_\mathrm{d}(\mathbf{r}) = \frac{1}{4\pi r^3}\left(3\hat{\mathbf{r}}(\hat{\mathbf{r}}\cdot\mathbf{m}) - \mathbf{m}\right) \tag{16}$$

where $\mathbf{r}$ is the vector displacement from the particle center with length r and direction $\hat{\mathbf{r}}$. The moment $\mathbf{m}$ represents the net magnetization of the particle material(s) obtained by integrating the local magnetization $\mathbf{M}(\mathbf{r})$ over the particle volume. In general, a particle's moment $\mathbf{m}$ depends on the presence of an external field $\mathbf{H}_\mathrm{e}$ and its history due to magnetic hysteresis. Figure S1b shows two qualitative magnetization curves for soft (blue) and hard (purple) magnetic materials as the external field is varied periodically in time. Under weak fields (shaded region), the behaviors of soft and hard materials are well approximated by two simple models for describing a particle's magnetic moment, which we refer to as the ferromagnetic model and the superparamagnetic model.

In the ferromagnetic model, the moment $\mathbf{m}$ is approximated as constant and does not depend on the external field. Examples of micro-particles described by this model include haematite cubes[S10–S12] and magnetic Janus spheres[S5–S9] (Fig. S1c). To avoid irreversible aggregation due to dipole-dipole interactions, magnetic particles are chosen or designed such that external fields H_e can disrupt particle aggregates. For spherical particles of diameter d, this condition implies that the magnetic moment m satisfies the inequality $m < 3.69 d^3 H_\mathrm{e}$, where the prefactor refers to the specific case of two spheres in a rotating field (see Section 3 below). For haematite cubes with a bulk magnetization of $M = 2.2 \times 10^3$ A/m, this conditions implies that particle aggregates can be disrupted by external fields of strength $H_\mathrm{e} = M/3.69 \approx 600$ A/m, which corresponds conveniently to that of air-core electromag-

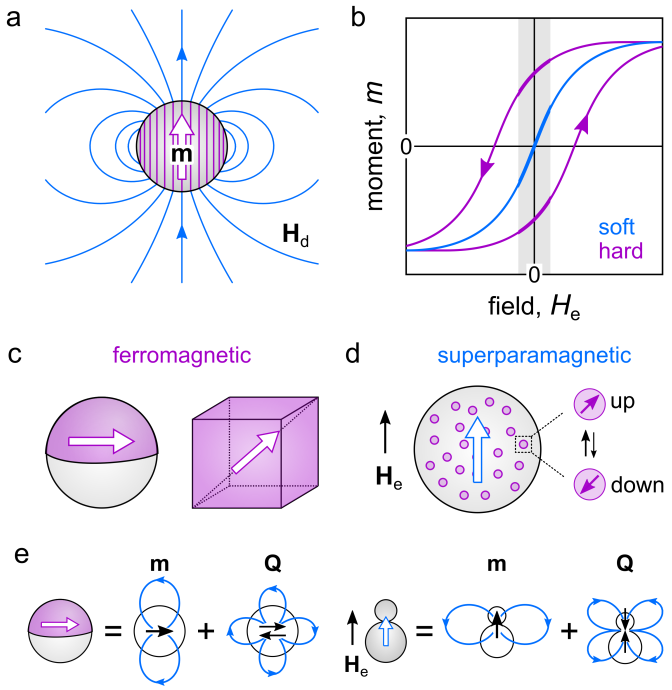

Figure S1: (a) Dipolar disturbance field $\mathbf{H}_\mathrm{d}$ due to a spherical particle of diameter d and uniform magnetization $\mathbf{M}$; the magnetic moment is $\mathbf{m} = \frac{1}{6}\pi d^3 \mathbf{M}$. (b) Magnetic hysteresis curves show the dependence of the magnetic moment m as a function of the external field strength H_e for soft and hard magnetic materials. The shaded region corresponds to weak fields, for which the ferromagnetic (m = constant) and superparamagnetic ($m \propto H_\mathrm{e}$) models are appropriate. (c) Examples of magnetic microparticles described by the ferromagnetic model: magnetic Janus spheres[S5–S9] (left) and haematite cubes[S10–S12] (right). (d) Example of a microparticle described by the superparamagnetic model: a polymer bead containing magnetic nanoparticles.[S13,S14] (e) The disturbance field surrounding asymmetric particles is described by a superposition of dipolar, quadrupolar, and higher order contributions (not shown).

netics. By contrast, for strongly magnetic materials like iron[S5,S7] and nickel[S6,S8,S9] (bulk magnetization, $M = 1.7$ and 0.5×10^6 A/m, respectively), only thin surface coatings are needed to create micron-scale Janus particles[S6,S8] with comparable magnetic moments of order $m \approx 3 \times 10^{-15}$ A m^2.

In the superparamagnetic model, the moment depends linearly on the external field as $\mathbf{m} = \boldsymbol{\alpha} \cdot \mathbf{H}_\mathrm{e}$, where $\boldsymbol{\alpha}$ is the magnetic polarizability tensor. Examples of microparticles described by this model include polystyrene beads containing iron oxide nanoparticles dispersed uniformly throughout their volume (e.g., Dynabeads;[S13–S15] Fig. S1d). Each nanopar-

ticle (diameter $d_{\rm np} \approx 10$ nm) contains a single magnetic domain in which the magnetization $\mathbf{M}_{\rm np}$ fluctuates "up" and "down" along the nanoparticle's easy axis. Within the polymer-nanoparticle composite the average magnetization is proportional to the applied field as $\mathbf{M} = \chi \mathbf{H}$, where the susceptibility χ depends on the volume fraction ϕ of magnetic solids as $\chi = \pi \mu_0 \phi M_{\rm np}^2 d_{\rm np}^3 / 18 k_{\rm B} T$ where $\mu_0 = 4\pi \times 10^{-7}$ N/A^2 is the vacuum permeability, and $k_{\rm B}T$ is the thermal energy.[S1] As the name implies, the susceptibility of *super*paramagnetic composites is many orders of magnitude larger than that of paramagnetic materials (cf. $\chi \approx 1.4$ for Dynabeads[S15] vs. $\chi \approx 5.7 \times 10^{-4}$ for 1 M holmium nitrate solutions[S16]). For a spherical particle in an isotropic medium with respective susceptibilities $\chi_{\rm p}$ and χ, the polarizability is proportional to the particle volume and the susceptibility contrast as $\alpha = \frac{1}{2}\pi d^3 (\chi - \chi_{\rm p})/(3 + 2\chi + \chi_{\rm p})$. In general, however, asymmetric particles exhibit different polarizabilities along the their principal axes as described by the symmetric tensor $\boldsymbol{\alpha}$.

Beyond the dipolar disturbance due to the magnetic moment, asymmetric particles contribute additional disturbances to the magnetic field characterized by higher order multipole moments (i.e., quadrupole, octapole, etc.). In particular, the quadrupole moment $\mathbf{Q}$ formed by the superposition of two antiparallel dipoles (either side-by-side or end-to-end) creates a disturbance field that decays as r^{-4} with distance from the particle center. Figure S1e illustrates the quadrupolar contribution to the disturbance field created by two asymmetric particles of different symmetries. Magnetic Janus spheres (Fig. S1e, left) are often characterized by a permanent magnetic moment oriented parallel to the Janus equator. An additional quadrupole contribution (defined relative to the particle center) shares the symmetry of the particle and describes how the dipole moment is displaced from the particle center, thereby influencing particle-particle interactions and self-assembly.[S5] Similarly, the magnetic polarization of an asymmetric, two-sphere dimer (Fig. S1e, right) creates an axially symmetric disturbance field as approximated by a superposition of dipolar and quadrupolar contributions.

2.2 Magnetic Forces and Torques

The magnetic torque $\mathbf{T}_\mathrm{m}$ on a particle with dipole $\mathbf{m}$ and quadrupole $\mathbf{Q}$ is related to the external field $\mathbf{B}_\mathrm{e}$ and its gradient evaluated at the particle center as

$$\mathbf{T}_\mathrm{m} = \mathbf{m} \times \mathbf{B}_\mathrm{e} + \tfrac{1}{3}(\mathbf{Q} \cdot \nabla) \times \mathbf{B}_\mathrm{e} + \dots \tag{17}$$

where additional contributions due to higher order moments and field gradients have been neglected (see Supporting Information).[S2,S3] Here, the magnetic induction field $\mathbf{B}$ (SI units of T or N/A m) is related to the magnetic field $\mathbf{H}$ as $\mathbf{B} = \mu_0(\mathbf{H} + \mathbf{M})$, which simplifies as $\mathbf{B} = \mu\mathbf{H}$ for linear magnetic media where $\mu = \mu_0(1 + \chi)$ is the permeability. In a spatially uniform field (no field gradients), the magnetic torque acts to align the dipole moment $\mathbf{m}$ parallel to the field (Fig. S2a). For micron-scale particles in 1 mT fields, the magnetic torque is ca. 3×10^{-18} N m, which enables particle rotation at angular speeds of $\Omega = mB_\mathrm{e}/\pi\eta d^3 \sim 900$ rad/s (140 Hz) in water with viscosity $\eta = 0.001$ Pa s. Importantly, the magnetic torque is much larger than the thermal energy at room temperature—that is, $mB_\mathrm{e}/k_\mathrm{B}T \sim 700 \gg 1$. As a result, Brownian motion can often be neglected in describing the dynamics of magnetically driven colloids.

While a uniform field acts to align a magnetic particle along a specified direction, it cannot control the particle orientation about the axis parallel its magnetic moment. By contrast, field gradients can be used to specify the orientation of a magnetic quadrupole in one of eight degenerate configurations in three dimensions (Fig. S2c).[S17] However, the magnitude of quadrupolar contributions to the torque are typically much smaller than those of the dipole. To see why, consider that the magnitude of the quadrupole is $Q \sim dm$ (unless special care is made to eliminate the dipole), and that of the field gradient is of order B_e/L, where L is a macroscopic length scale over which the field varies. The ratio of the quadrupolar and dipolar torques is of order $d/L \sim 10^{-4} \ll 1$, when $d \sim 1$ μm and $L \sim 1$ cm. Consequently, the magnetic torque is well approximated by the leading order contribution

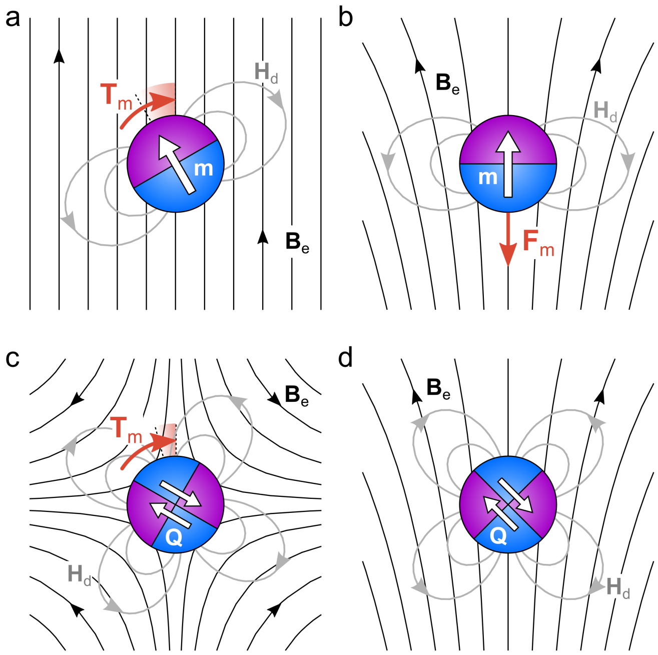

Figure S2: (a) A uniform field $\mathbf{B}_\mathrm{e}$ exerts a magnetic torque $\mathbf{T}_\mathrm{m}$ on a particle as to orient its magnetic moment $\mathbf{m}$ parallel with the field. Upon rotation, the disturbance field $\mathbf{H}_\mathrm{d}$ partially cancels the external field $\mathbf{B}_\mathrm{e}$ thereby lowering the magnetic energy density $\frac{1}{2}\mathbf{H}\cdot\mathbf{B}$.[S4] (b) A field gradient $\nabla\mathbf{B}_\mathrm{e}$ exerts a magnetic force $\mathbf{F}_\mathrm{m}$ on the field-aligned particle directed to regions of higher field strength. (c) A field gradients exert a torque on a magnetic quadrupole $\mathbf{Q}$ formed by two antiparallel dipoles displaced perpendicular to the dipole direction. The particle adopts one of eight stable orientations in the field. (d) Addition of a uniform field to the field gradient in (c) does not alter the final orientation of the quadrupolar particle.

in equation (17).

The magnetic force $\mathbf{F}_\mathrm{m}$ on a particle with dipole $\mathbf{m}$ and quadrupole $\mathbf{Q}$ in an external field $\mathbf{B}_\mathrm{e}(\mathbf{r})$ is given by

$$\mathbf{F}_\mathrm{m} = \mathbf{m}\cdot\nabla\mathbf{B}_\mathrm{e} + \tfrac{1}{6}\mathbf{Q} : \nabla\nabla\mathbf{B}_\mathrm{e} + \ldots \tag{18}$$

Importantly, there is no force on a magnetic particle in a spatially uniform magnetic field. Instead, the leading order contribution is proportional to the field gradient: magnetic particles rotate to align their moment with the external field and translate towards regions of higher field strength (Fig. S2b). According to Earnshaw's theorem, it is not possible to stably position a magnetic particle in an external field as the particle will invariably move towards the field source or sink (e.g., a permanent magnetic or an electromagnetic coil).[S18]

A notable exception occurs when the permeability of the particle μ_{p} is less than that of the surrounding medium μ resulting in a negative polarizability $\alpha \propto (\mu_{\mathrm{p}} - \mu) < 0$. In that case, the leading order contribution to the force, $\mathbf{F}_{\mathrm{m}} = \frac{1}{2}\mu\alpha\nabla H_{\mathrm{e}}^{2}$, is directed to regions of low field strength thereby enabling magnetic levitation.

3 Two Ferromagnetic Spheres in a Rotating Field

We consider two identical spheres with diameter d and magnetic moment $\mathbf{m}$ in an unbounded fluid of viscosity η. Guided by dipole-dipole interactions, the spheres approach contact and their moments align (head-to-tail) parallel to each other and to the line of centers. Upon application of a rotating magnetic field of magnitude B_e and frequency ω, the spheres can either rotate together as a rigid body or about their respective centers thereby disrupting the two-sphere assembly. Here, we consider a hydrodynamic model of particle dynamics at low Reynolds number and identify the critical field strength H_e^* required to break the dipole-dipole interaction between the two spheres.

Magnetic Force and Torque

The magnetic torques on spheres 1 and 2 due to the external field and the dipolar disturbance fields are given by

$$\mathbf{T}_1 = \mathbf{m}_1 \times \mathbf{B}(\mathbf{x}_1) = \mathbf{m}_1 \times \left(\mathbf{B}_\mathrm{e}(t) + \frac{\mu_0}{4\pi} \left(\frac{3\mathbf{r}(\mathbf{r} \cdot \mathbf{m}_2)}{r^5} - \frac{\mathbf{m}_2}{r^3} \right) \right) \tag{19}$$

$$\mathbf{T}_2 = \mathbf{m}_2 \times \mathbf{B}(\mathbf{x}_2) = \mathbf{T}_1 \tag{20}$$

where $\mathbf{r} = \mathbf{x}_2 - \mathbf{x}_1$ is the displacement vector directed from sphere 1 to sphere 2. The magnetic forces on spheres 1 and 2 due to their dipole-dipole interactions are

$$\begin{aligned} \mathbf{F}_1 = \mathbf{m}_1 \cdot \nabla \mathbf{B}(\mathbf{x}_1) = \frac{\mu_0}{4\pi} \bigg(& \frac{15(\mathbf{r} \cdot \mathbf{m}_1)(\mathbf{r} \cdot \mathbf{m}_2)\mathbf{r}}{r^7} - \frac{3(\mathbf{m}_1 \cdot \mathbf{m}_2)\mathbf{r}}{r^5} \\ & - \frac{3(\mathbf{r} \cdot \mathbf{m}_2)\mathbf{m}_1}{r^5} - \frac{3(\mathbf{r} \cdot \mathbf{m}_1)\mathbf{m}_2}{r^5} \bigg) \end{aligned} \tag{21}$$

$$\mathbf{F}_2 = \mathbf{m}_2 \cdot \nabla \mathbf{B}(\mathbf{x}_2) = -\mathbf{F}_1 \tag{22}$$

Hydrodynamics

At low Reynolds number, the linear and angular velocities of the two spheres through a quiescent fluid are related to the magnetic forces and torques by the hydrodynamic resistance tensor as

$$\begin{pmatrix} \mathbf{F}_1 \\ \mathbf{F}_2 \\ \mathbf{T}_1 \\ \mathbf{T}_2 \end{pmatrix} = \begin{pmatrix} \mathbf{A}_{11} & \mathbf{A}_{12} & \tilde{\mathbf{B}}_{11} & \tilde{\mathbf{B}}_{12} \\ \mathbf{A}_{12} & \mathbf{A}_{22} & \tilde{\mathbf{B}}_{11} & \tilde{\mathbf{B}}_{12} \\ \mathbf{B}_{11} & \mathbf{B}_{12} & \mathbf{C}_{11} & \mathbf{C}_{12} \\ \mathbf{B}_{12} & \mathbf{B}_{22} & \mathbf{C}_{21} & \mathbf{C}_{22} \end{pmatrix} \cdot \begin{pmatrix} \mathbf{U}_1 \\ \mathbf{U}_2 \\ \mathbf{\Omega}_1 \\ \mathbf{\Omega}_2 \end{pmatrix} \tag{23}$$

For two nearly touching spheres, the components of the resistance tensor are known (see, for example, Kim & Karrila[S19]). Together with equations (19)–(20) for the torques and equations (21)–(22) for the forces, equation (23) describes the rotational and translational dynamics of the two spheres in the time-varying field $\mathbf{B}_\mathrm{e}(t)$.

Stability of Rigid-Body Rotation

For sufficiently high field strengths B_e and low frequencies ω (to be determined), the assembly of two contacting spheres can rotate together as a rigid body with angular velocity equal to that of the external field

$$\mathbf{\Omega}_1 = \mathbf{\Omega}_2 = \boldsymbol{\omega} \tag{24}$$

The linear velocities of the two spheres with respect to an origin at the point of contact are

$$\mathbf{U}_1 = -\frac{1}{2}\,\boldsymbol{\omega} \times \mathbf{r} \quad \text{and} \quad \mathbf{U}_2 = \frac{1}{2}\,\boldsymbol{\omega} \times \mathbf{r} \tag{25}$$

The force and torque on sphere 1 are related to the driving frequency as

$$\mathbf{F}_1 = -(1.062)\pi\eta d(\boldsymbol{\omega} \times \mathbf{d}) \tag{26}$$

$$\mathbf{T}_1 = (0.676)\pi\eta d^3\boldsymbol{\omega} \tag{27}$$

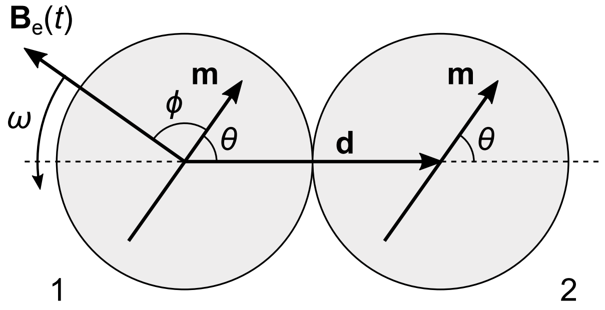

Figure S3: Two spheres with diameter d and moment $\mathbf{m}$ moving in a rotating magnetic field of strength B_e and frequency ω.

where $\mathbf{d}$ is the displacement of length d directed from sphere 1 to sphere 2 (i.e., $\mathbf{d} = \mathbf{r}$ for contacting spheres), and the numeric prefactors are given by lubrication theory.[S19] For parallel dipoles in the plane of the rotating field, the magnetic torque on sphere 1 acts along the direction of rotation $\hat{\boldsymbol{\omega}}$ with magnitude

$$\mathbf{T}_1 = \left(m B_\mathrm{e} \sin\phi - \frac{3\mu_0 m^2 \sin\theta\cos\theta}{4\pi d^3} \right) \hat{\boldsymbol{\omega}} \tag{28}$$

where ϕ is the angle between the moment $\mathbf{m}$ and the field $\mathbf{B}_\mathrm{e}$, and θ is the angle between the moment $\mathbf{m}$ and the displacement vector $\mathbf{d}$ (Fig. S3). The magnetic force on sphere 1 has components parallel and perpendicular to the line of centers

$$\mathbf{F}_1 = \frac{3\mu_0 m^2 (3\cos^2\theta - 1)}{4\pi d^4} \hat{\mathbf{d}} - \frac{6\mu_0 m^2 \sin\theta\cos\theta}{4\pi d^4} \hat{\boldsymbol{\omega}} \times \hat{\mathbf{d}} \tag{29}$$

Equating expressions (26) and (29) for the force and (27) and (28) for the torque, one can solve for the angles θ and ϕ for a given field strength H_e and rotation frequency ω. When solutions exists, their stability requires that

$$\hat{\mathbf{d}} \cdot \mathbf{F}_1 > 0 \quad \text{such that} \quad \theta < \arccos(1/\sqrt{3}) \approx 0.955 \tag{30}$$

$$\partial_\theta(\mathbf{T}_1 \cdot \hat{\boldsymbol{\omega}}) < 0 \quad \text{such that} \quad \theta < \pi/4 \approx 0.785 \tag{31}$$

$$\partial_\phi(\mathbf{T}_1 \cdot \hat{\boldsymbol{\omega}}) > 0 \quad \text{such that} \quad \phi < \pi/2 \approx 1.57 \tag{32}$$

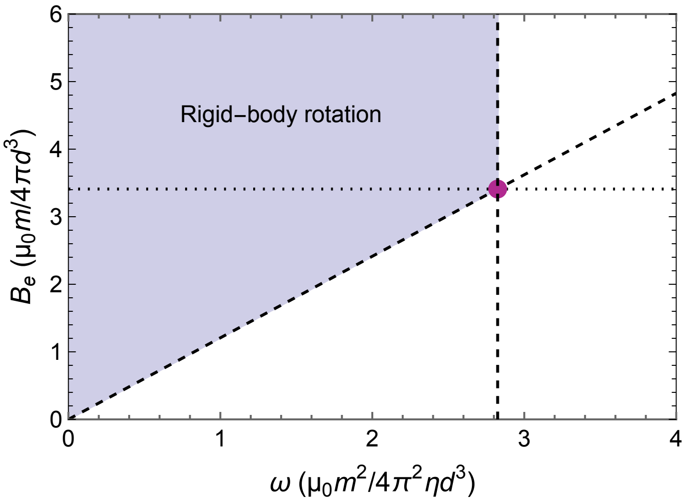

Figure S4: Field-frequency parameter space showing the conditions (shaded region) required for stable rigid-body rotation of two ferromagnetic spheres in a rotating field. The external field is scaled by the dipole field strength $\mu_0 m/4\pi d^3$; the rotation frequency is scaled by the characteristic relaxation rate $\mu_0 m^2/4\pi^2\eta d^3$. The critical field strength required to break the dipole-dipole interactions is $B_\mathrm{e}^* = (3.410)\mu_0 m/4\pi d^3$.

Here, the first expression indicates that the dipole-dipole interaction between the two spheres must be attractive. Under these conditions, magnetic attraction is balanced by short ranged repulsive forces (not by hydrodynamic forces). The second and third expressions indicate that perturbations to the angles θ and ϕ that describe the particle orientation must decay (not grow) in time.

Figure S4 shows the region of the field-frequency parameter space where rigid-body rotation is stable. A critical field strength $B_\mathrm{e}^* = (3.410)\mu_0 m/4\pi d^3$ separates two distinct mechanisms by which rigid-body rotation becomes unstable with increasing frequency. At low field strength ($B_\mathrm{e} < B_\mathrm{e}^*$), rigid-body rotation becomes unstable due to violation of equation (32). Above a critical frequency $\omega^* = (0.829)mB_\mathrm{e}/\pi\eta d^3$, the magnetic torque is insufficient to drive particle rotation at the speed of the driving field. Nevertheless, the dipole-dipole interactions holding the spheres together remain unbroken. At high field strength ($B_\mathrm{e} > B_\mathrm{e}^*$), rigid-body rotation becomes unstable due to violation of equation (31). Above a critical frequency $\omega^* = (2.82)\mu_0 m^2/4\pi^2\eta d^3$, the dipole-dipole interactions are broken, and the spheres rotate instead about their respective centers.

4 Magnetic Rollers in a Rotating Field

4.1 Ferromagnetic Rollers in a Rotating Field

Here, we consider the dynamics of a ferromagnetic sphere in a rotating magnetic field of the form

$$\mathbf{B}(t) = B\big(\cos(\omega t)\mathbf{e}_y + \sin(\omega t)\mathbf{e}_z\big) \tag{33}$$

The field rotates in the $\mathbf{e}_x$ direction with frequency ω and magnitude B. The magnetic torque on the sphere is

$$\mathbf{T} = \mathbf{m} \times \mathbf{B} \tag{34}$$

where $\mathbf{m}$ is the magnetic moment, which is constant in the particle reference frame. Assuming that the moment lies in the yz plane, it can be parameterized by a single angle φ as

$$\mathbf{m} = m\big(\cos(\varphi)\mathbf{e}_y + \sin(\varphi)\mathbf{e}_z\big) \tag{35}$$

The magnetic torque on the sphere can therefore be written as

$$\mathbf{T} = mB\sin(\omega t - \varphi)\mathbf{e}_x \tag{36}$$

This torque is balanced by the hydrodynamic resistance to rotation, which is linearly proportional to the angular velocity $\boldsymbol{\Omega}$

$$\boldsymbol{\Omega} = \dot{\varphi}\,\mathbf{e}_x = \frac{mB}{\lambda}\sin(\omega t - \varphi)\mathbf{e}_x \tag{37}$$

where λ is a resistance coefficient.[S20] When the sphere is positioned above a solid wall, the field induced rotation in the x-direction drives particle translation in the negative y-direction as

$$\mathbf{U} = -\kappa a \Omega \mathbf{e}_y \tag{38}$$

where $0 < \kappa < \frac{1}{4}$ is the hydrodynamic traction.[S20]

The dynamics of equation (37) is characterized by a single dimensionless parameter $\omega\lambda/mB$, which describes the ratio between the driving frequency ω and the relaxation rate mB/λ. When this parameter is small, the particle rotates in lock-step with the rotating field

$$\Omega = \omega \quad \text{for} \quad \omega < \omega_\mathrm{c} = \frac{mB}{\lambda} \tag{39}$$

At higher frequencies, the angular velocity oscillates with a frequency of $\sqrt{\omega^2 - \omega_\mathrm{c}^2}$ about a non-zero value

$$\langle \Omega \rangle = \omega - \sqrt{\omega^2 - \omega_c^2} \quad \text{for} \quad \omega > \omega_\mathrm{c} \tag{40}$$

Here, ω_c is a critical frequency—sometimes called the step-out frequency—that describes the transition between synchronous and asynchronous rotation in the driving field.

4.2 Superparamagnetic Rollers in a Rotating Field

We now consider the case of a superparamagnetic sphere with a magnetic moment $\mathbf{m}$ that relaxes to its equilibrium value $\alpha\mathbf{B}$ with a characteristic time scale τ. The moment evolves in time as

$$\dot{\mathbf{m}} = -\frac{1}{\tau}\big(\mathbf{m} - \alpha\mathbf{B}(t)\big) + \boldsymbol{\Omega} \times \mathbf{m} \tag{41}$$

where α is the magnetic polarizability of the particle. For rotation in the yz plane, we can parameterize the magnetic moment as

$$\mathbf{m} = m(\cos\phi\,\mathbf{e}_y + \sin\phi\,\mathbf{e}_z) \tag{42}$$

In a rotating field (33), the balance of magnetic and hydrodynamic torques implies the following expression for the angular velocity in the x-direction

$$\Omega = \dot{\varphi} = \frac{mB}{\lambda}\sin(\omega t - \phi) \tag{43}$$

Substituting this result into equation (41), the dynamics of the moment magnitude m and orientation ϕ can be expressed as

$$\dot{m} = -\frac{1}{\tau}\big(m - \alpha B \cos(\omega t - \phi)\big) \tag{44}$$

$$\dot{\phi} = \left(\frac{mB}{\lambda} + \frac{\alpha B}{m\tau}\right) \sin(\omega t - \phi) \tag{45}$$

The dynamics is characterized by three time scales: the internal relaxation rate τ^{-1}, the external relaxation rate $\alpha B^2/\lambda$, and the driving frequency ω. We focus on the limiting regime in which the internal relaxation rate is much faster than the external relaxation rate $\tau\alpha B^2/\lambda \ll 1$. Under these conditions, there exists a stable rotating solution of the form $\phi(t) = \omega t - c$ such that

$$m = \alpha B \cos c \tag{46}$$

$$\omega = \frac{1}{\tau}\tan c + \frac{\alpha B^2}{\lambda}\sin c \cos c \approx \frac{1}{\tau}\tan c \tag{47}$$

Substituting this result into equation (43), we obtain the following approximation for the angular velocity

$$\Omega = \frac{\alpha B^2}{\lambda}\frac{\tau\omega}{1 + (\tau\omega)^2} \tag{48}$$

References

(S1) Rosensweig, R. E. *Ferrohydrodynamics*; Courier Corporation, 2013.

(S2) Jackson, J. D. *Classical electrodynamics*, 3rd ed.; John Wiley & Sons, Inc., 1999.

(S3) Jones, T. B.; Washizu, M. Multipolar dielectrophoretic and electrorotation theory. *J. Electrostat.* **1996**, *37*, 121–134.

(S4) Landau, L. D.; Lifshitz, E. *Electrodynamics of continuous media*; Pergamon Press, 1984; Vol. 8.

(S5) Smoukov, S. K.; Gangwal, S.; Marquez, M.; Velev, O. D. Reconfigurable responsive structures assembled from magnetic Janus particles. *Soft Matter* **2009**, *5*, 1285–1292.

(S6) Sinn, I.; Kinnunen, P.; Pei, S. N.; Clarke, R.; McNaughton, B. H.; Kopelman, R. Magnetically uniform and tunable Janus particles. *Appl. Phys. Lett.* **2011**, *98*, 024101.

(S7) Ren, B.; Ruditskiy, A.; Song, J. H.; Kretzschmar, I. Assembly behavior of iron oxide-capped Janus particles in a magnetic field. *Langmuir* **2012**, *28*, 1149–1156.

(S8) Yan, J.; Bae, S. C.; Granick, S. Rotating crystals of magnetic Janus colloids. *Soft Matter* **2015**, *11*, 147–153.

(S9) Fei, W.; Driscoll, M. M.; Chaikin, P. M.; Bishop, K. J. M. Magneto-capillary dynamics of amphiphilic Janus particles at curved liquid interfaces. *Soft Matter* **2018**, *14*, 4661–4665.

(S10) Sacanna, S.; Rossi, L.; Pine, D. J. Magnetic click colloidal assembly. *J. Am. Chem. Soc.* **2012**, *134*, 6112–6115.

(S11) Driscoll, M.; Delmotte, B.; Youssef, M.; Sacanna, S.; Donev, A.; Chaikin, P. Unstable fronts and motile structures formed by microrollers. *Nat. Phys.* **2017**, *13*, 375–379.

(S12) Soni, V.; Bililign, E. S.; Magkiriadou, S.; Sacanna, S.; Bartolo, D.; Shelley, M. J.; Irvine, W. T. The odd free surface flows of a colloidal chiral fluid. *Nat. Phys.* **2019**, *15*, 1188–1194.

(S13) Martinez-Pedrero, F.; Ortiz-Ambriz, A.; Pagonabarraga, I.; Tierno, P. Colloidal microworms propelling via a cooperative hydrodynamic conveyor belt. *Phys. Rev. Lett.* **2015**, *115*, 138301.

(S14) Byrom, J.; Han, P.; Savory, M.; Biswal, S. L. Directing assembly of DNA-coated colloids with magnetic fields to generate rigid, semiflexible, and flexible chains. *Langmuir* **2014**, *30*, 9045–9052.

(S15) Lobmeyer, D. M.; Biswal, S. L. Grain boundary dynamics driven by magnetically induced circulation at the void interface of 2D colloidal crystals. *Sci. Adv.* **2022**, *8*, eabn5715.

(S16) Timonen, J. V.; Grzybowski, B. A. Tweezing of magnetic and non-magnetic objects with magnetic fields. *Adv. Mater.* **2017**, *29*, 1603516.

(S17) Diller, E.; Giltinan, J.; Lum, G. Z.; Ye, Z.; Sitti, M. Six-Degree-of-Freedom Magnetic Actuation for Wireless Microrobotics. *The International Journal of Robotics Research* **2016**, *35*, 114–128.

(S18) Simon, M.; Geim, A. Diamagnetic levitation: Flying frogs and floating magnets. *J. Appl. Phys.* **2000**, *87*, 6200–6204.

(S19) Kim, S.; Karrila, S. J. *Microhydrodynamics: Principles and selected applications*; Dover Publications, 2005.

(S20) Goldman, A. J.; Cox, R. G.; Brenner, H. Slow viscous motion of a sphere parallel to a plane wallI Motion through a quiescent fluid. *Chem. Eng. Sci.* **1967**, *22*, 637–651.
